# Targeting a Novel AREG ^+^ Dendritic Cell Alleviates Renal Fibrosis Induced by Ischemia–Reperfusion Injury

**DOI:** 10.1111/cpr.70287

**Published:** 2026-10-05

**Authors:** Xin Luo, Longbin Xiong, Zhaohui Zhou, Lihao Zhang, Yulu Peng, Xiangpeng Zou, Yixin Huang, Yisong Lin, Xiaofeng Yang, Zhanji Zhan, Fangjian Zhou, Youhua Liu, Chunping Yu, Baokui Ye, Zhiling Zhang

**Affiliations:** ^1^ Departments of Urology Sun Yat‐sen University Cancer Center Guangzhou China; ^2^ State Key Laboratory of Oncology in Southern China, Collaborative Innovation Center for Cancer Medicine Guangzhou China; ^3^ Guangdong Provincial Clinical Research Center for Cancer; State Key Laboratory of Oncology in Southern China Guangzhou China; ^4^ State Key Laboratory of Organ Failure Research, Division of Nephrology Nanfang Hospital, Southern Medical University Guangzhou China; ^5^ Department of Urology, Minimally Invasive Surgery Center The First Affiliated Hospital of Guangzhou Medical University Guangzhou China; ^6^ Department of Intensive Care Unit Sun Yat‐sen University Cancer Center Guangzhou China

**Keywords:** epithelial mesenchymal transition, human ischemia–reperfusion injury, kidney fibrosis, radical nephrectomy, single cell sequencing

## Abstract

Renal ischemia–reperfusion injury (RIRI) is a major cause of acute tubular damage and a key driver of maladaptive repair and fibrosis. However, the cellular initiators that connect early immune activation to tubular epithelial–mesenchymal transition (EMT) during RIRI remain poorly defined. Here, we established a human RIRI model by modifying the surgical procedures in patients undergoing radical nephrectomy and confirmed successful injury induction by tubular dilation, severe mitochondrial swelling and increased apoptosis in renal tissues. Using single‐cell RNA sequencing, we uncovered an underappreciated dendritic cell (DC) subset, CD11c^+^AREG^+^ DCs, that expands after RIRI, coinciding with EMT activation in tubular epithelial cells at early stages. Notably, ligand–receptor inference revealed markedly enhanced communication between CD11c^+^AREG^+^ DCs and tubular epithelial cells after RIRI, suggesting the AREG–EGFR as a dominant axis. Mechanistically, CD11c^+^AREG^+^ DCs initiate and promote tubular EMT via AREG‐dependent EGFR signalling, establishing a direct causal link between this DC population and injured tubular epithelial cells. Importantly, Areg knockdown or DC depletion significantly alleviated tubular damage and fibrosis and preserved kidney function in the IRI mouse model, which highlights these cells could be potential therapeutic targets for mitigating RIRI.

## Graphical abstract

Renal IRI induces tubular EMT and fibrosis. CD11c^+^AREG^+^ DCs mediate the process via AREG‐EGFR axis, serving as promising therapeutic targets.

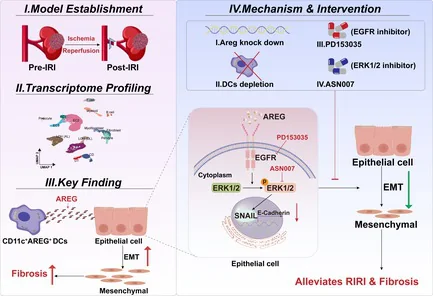

## 1 Introduction

Renal ischemia–reperfusion injury (RIRI) results when blood flow is restored following a period of ischemia, causing tissue damage [1]. It is a common pathological process accompanying shock, cardiac surgery and renal surgery, often culminating in acute kidney injury (AKI) [2]. Studies have shown that AKI increases the risk of chronic kidney disease (CKD), shortening survival time and reducing quality of life [3]. Globally, AKI affects approximately 21.6% of adult inpatients, with a mortality rate reaching 23.9% [4]. Treatments like renal replacement therapy impose a significant socioeconomic burden [5]. Thus, understanding the mechanisms of RIRI is crucial to prevent AKI and its progression to CKD.

Many studies have investigated the pathogenesis of RIRI using animal model [6, 7]. However, these models often failed to accurately predict human outcomes due to interspecies physiological differences [8]. Thus, many potential therapeutic strategies effective in animal models failed to demonstrate clinical benefit in humans. For example, although Atrial Natriuretic Peptide (ANP) and Insulin‐like Growth Factor‐1 (IGF‐1) ameliorated AKI in preclinical models, multicentre clinical trials revealed no significant improvement in renal function or dialysis requirements in patients [9, 10]. These discrepancies underscore the urgent need for human‐derived RIRI data to better elucidate its mechanisms and guide therapeutic innovation. While previous studies utilizing kidney biopsy specimens from AKI patients have provided valuable insights into the disease's pathophysiology, paired kidney samples obtained before and after RIRI remain exceedingly rare, despite their potential to reveal early mechanistic events that initiate renal injury.

Partial nephrectomy (PN) represents an ideal human model for RIRI research, as temporary clamping of the renal artery is commonly performed to minimize intraoperative bleeding [11]. Our previous research demonstrated that renal parenchymal fibrosis progresses after PN [12] and the degree of RIRI closely correlates with parenchymal atrophy and functional decline of the operated kidney [13, 14]. Although ischemia during PN is typically less severe than that observed in other clinical settings, it offers a unique opportunity to study controlled ischemia–reperfusion events directly in human renal tissue.

Parekh and colleagues performed renal biopsy before and 5 min after ischemia during PN but observed minimal histological injury, likely due to the short ischemia (median 32 min) and reperfusion (5 min) times [15]. Park et al. performed bulk RNA sequencing on paired renal tissue samples from PN patients, revealing global transcriptional shifts but lacking cell‐type resolution necessary to dissect specific molecular and cellular responses [16].

Preclinical evidence suggests that renal immune activation occurs early in the course of RIRI. In particular, dendritic cells (DCs) orchestrate the local immune response by secreting cytokines and chemokines that stimulate the neighbouring renal parenchymal cells and intrinsic immune cells, ultimately driving inflammation and interstitial fibrosis [17, 18, 19, 20, 21].

In the present study, we established a human RIRI model in patients who underwent radical nephrectomy (RN) by clamping the renal artery for 46 min (median) and then unclamping for 28 min (median) before artery transection and kidney removal. Tissue samples were obtained before ischemia and after ischemia/reperfusion. Using scRNA‐seq and bulk‐RNA‐seq, we comprehensively characterized cellular composition, gene expression and intercellular communication in human kidneys subjected to ischemia–reperfusion injury (IRI). To our knowledge, this represents the first study to define a human RIRI transcriptomic landscape at single‐cell resolution. Our findings identify a novel subset of CD11c^+^Amphiregulin^+^ (AREG^+^) dendritic cells, which appear to mediate early inflammation and trigger epithelial–mesenchymal transition (EMT) in renal tubular epithelial cells—an early and pivotal event leading to renal fibrosis.

## 2 Methods

### 2.1 Patients and Ethical Approval

Twenty‐one patients scheduled for RN due to renal cell carcinoma (RCC) were enrolled in this study (Table S1). The study was conducted in accordance with the ethical standards of the Declaration of Helsinki and the institutional guidelines for medical research involving human subjects. The human protocol was approved by the Institutional Review Board of Sun Yat‐sen University Cancer Center (SYSUCC; approval No. B2023‐162‐01) and all participants provided written informed consent prior to inclusion. The additional ischemia/reperfusion manipulations were performed exclusively on kidneys that were already scheduled for surgical removal and no deviation from the standard of care occurred apart from the planned biopsy and the time‐limited vascular clamping. Detailed descriptions of the risk–benefit evaluation, intraoperative safety measures and postoperative adverse‐event monitoring are provided in the Supporting Information Methods and Table S2.

### 2.2 Human RIRI Model

Adults scheduled to undergo RN for RCC were considered eligible for IRI modelling. Inclusion criteria required normal preoperative renal function and no history of diabetes, hypertension or other systemic diseases. Patients with pre‐existing renal impairment, multiple comorbidities or intraoperative hemodynamic instability were excluded. Conventional RN usually involves the following steps: (1) exposing the kidney and retroperitoneal great vessels; (2) separating, clipping and transecting the renal artery; (3) exposing the kidney's periphery; and (4) removing lymph nodes adjacent to the great vessels [22]. For the IRI model, we clamped the renal artery with bulldog forceps after the exposure of the renal artery; at this time, the kidney enters an ischemic state. Then we mobilize the entire kidney and free the ureter and renal vein in preparation for transection. Next, we release the renal artery, allowing the kidney to enter a reperfusion state. We then proceed with lymph node dissection. After completing the lymph node dissection, we transect the renal artery, vein and ureter and remove the kidney. Biopsies were taken before clamping and after kidney removal at a site remote from the tumour (Figure 1C). Surgery time and blood loss were compared between IRI and regular nephrectomy patients to assess the safety of the IRI modelling. Further details of the methods are displayed in Supporting Information Methods.

**FIGURE 1:**
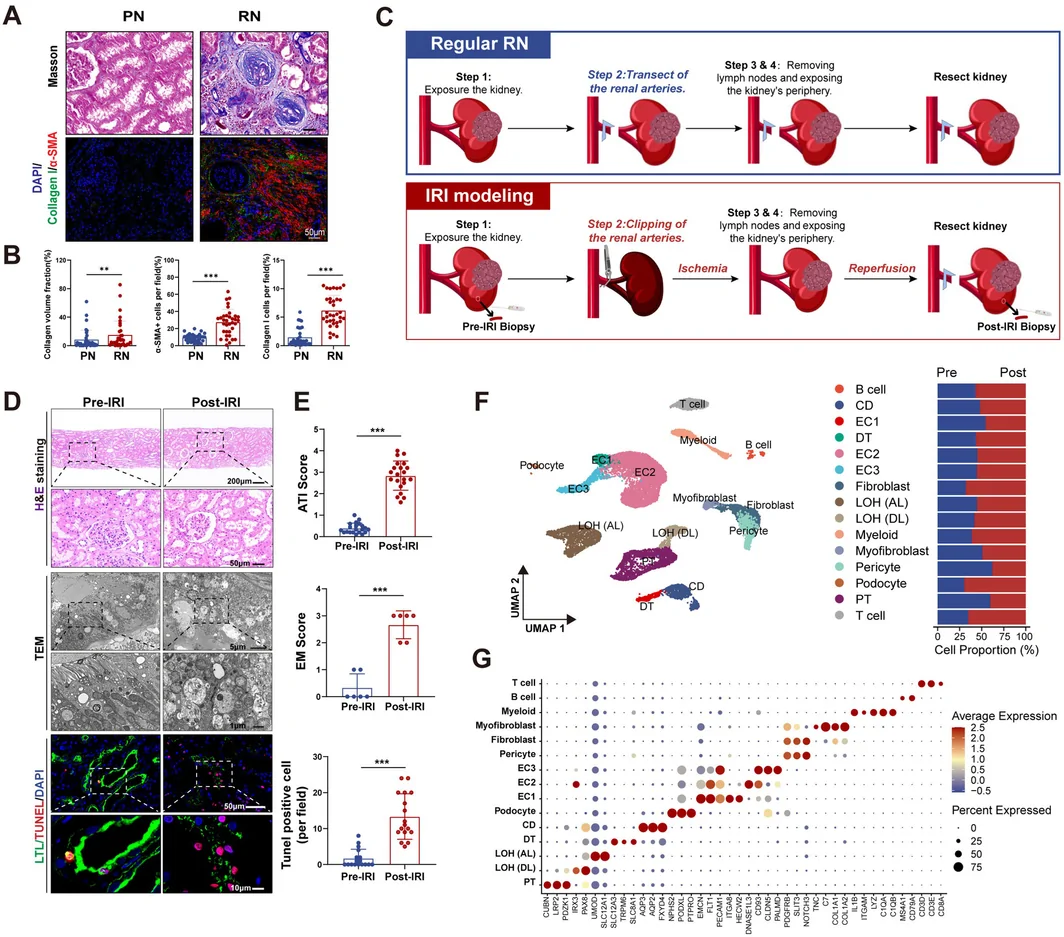
Comprehensive histological and single‐cell RNA sequencing analysis in human renal ischemia/reperfusion injury model. (A and B) Masson staining indicates increased fibrosis in RN versus PN samples, highlighted in immunofluorescent micrographs (collagen I in green, α‐SMA in red, nuclei in blue, scale bar = 100 μm); bar charts detail differences in collagen volume and cell counts (*n* = 35). (C) Schematic of regular radical nephrectomy and IRI modelling in humans. (D and E) H&E staining (scale bars: 200, 50 μm) and TUNEL assays (scale bars: 50, 10 μm) pre‐ and post‐IRI; ultrastructural changes shown in the middle panel (scale bars: 5 μm low mag., 1 μm high mag.). Bar Charts show acute tubular injury (ATI) scores, electron microscopy (EM) scores and TUNEL^+^ cell counts in pre‐ and post‐IRI groups (ATI, EM: *n* = 21, 6; TUNEL^+^: *n* = 17). (F and G) UMAP plot identifies cell clusters by gene expression variability; histograms show cell type percentages across groups; dot plot details canonical gene expression by cell type. **p < 0.01; ***p < 0.001.

## 3 Results

### 3.1 Establishment of Human Renal Ischemia–Reperfusion Model

Previous studies have shown that RIRI during PN can significantly impair kidney function (Table S3). To validate these findings while minimizing confounding factors such as diabetes and hypertension, we retrospectively analysed PN patients from SYSUCC with normal preoperative renal function and no underlying diseases. Longer ischemia duration was correlated with higher acute ipsilateral renal dysfunction (AIRD) spectrum score and greater loss of ipsilateral renal parenchymal volume (Figure S1), which was further associated with progressive renal fibrosis during long‐term follow‐up (Figure 1A,B).

To comprehensively characterize the renal pathological alterations and delineate the mechanisms driving long‐term fibrosis specifically attributable to RIRI in humans, we established a human RIRI model using patients without underlying diseases or pre‐existing renal impairment (ischemia time: 46 min [IQR: 32–55]; reperfusion time: 28 min [IQR: 19–33]), as described in the Methods section (Figure 1C, Table S2). All patients selected for RIRI modelling were clinically stable and free of complications preoperatively, intraoperatively and postoperatively. Additionally, operative time and blood loss were comparable to those in the regular RN group (Table S3). Severe renal injury, particularly in the corticomedullary junction post‐ischemia–reperfusion, was observed via H&E staining (tubular dilation, epithelial flattening and loss of brush borders with occasional sloughed cells), electron microscopy (mitochondrial swelling) and increased tubular cell apoptosis (Figure 1D,E). These findings confirm the successful establishment of the human RIRI model.

### 3.2 scRNA‐Seq Identifies Kidney Biopsy Cell Types

Given that ischemia duration varied among patients, we selected three representative cases with relatively prolonged ischemia to better reflect RIRI‐induced renal injury for subsequent mechanistic analyses. After quality control and batch effect removal between samples, 22,163 single cells were clustered into 25 major clusters using the Uniform manifold approximation and projection (UMAP) method. Cluster‐specific genes were used to annotate cell types with classic markers described in previous studies [23, 24, 25, 26, 27]. We identified five types of epithelial cells in five clusters, including PT (CUBN^+^, LRP2^+^, PDZK1^+^), LOH(DL) (IRX3^+^, PAX8^+^), LOH(AL) (UMOD^+^, SLC12A1^+^), DT (SLC12A3^+^, TRPM6^+^, SLC8A1^+^) and CD (AQP3^+^, AQP2^+^, FXYD4^+^). Three types of immune cells were found in eight clusters: T (CD3D^+^, CD3E^+^, CD8A^+^), B (MS4A1^+^, CD79A^+^), Myeloid (IL1B^+^, ITGAM^+^, LYZ^+^, C1QA^+^, C1QB^+^). Three types of endothelial cells (EMCN^+^, FLT1^+^, PECAM1^+^) were identified, including EC1 (ITGA8^+^, HECW2^+^), EC2 (DNASE1L3^+^) and EC3 (CD93^+^, CLDN5^+^, PALMD^+^); fibroblasts (PDGFRB^+^, SLIT3^+^, NOTCH3^+^, COL1A1^+^ and COL1A2^+^), myofibroblast(COL1A2^+^)and pericyte (PDGFRB^+^, SLIT3^+^, NOTCH3^+^) (Figure 1F,G, Figure S2 and Table S4).

### 3.3 scRNA‐Seq Reveals Damaged Subpopulations of Proximal Tubule Cells

Proximal tubule cell populations were identified using unsupervised clustering, including PT‐C1 (normal proximal tubule) and PT‐C2 (injured proximal tubule) (Figure 2A). Our results demonstrated that genes (VCAM1, ITGAV, AKAP12, CDKN1A, TGFBR2) associated with the failed repair state of the tubule were highly expressed in PT‐C2 (Figure 2B) [28]. Functional enrichment GSEA analysis showed the top three pathways upregulated in PT‐C1 and PT‐C2 after IRI, all of which included the EMT pathway (Figure 2C,D). To further investigate proximal tubule injury responses, we conducted cell trajectory analysis using pseudotime analysis to reconstitute the biopsy proximal tubule clusters. We observed a smooth transition between PT‐C1 and PT‐C2 (Figure 2E–G). Gene clustering with a pseudotemporal expression pattern was organized into four clusters. Genes in clusters 2 and 4, including CDKN1A, SEMA5A, VCAM1, CCL2, VIM, S100A2, CXCL2, NFKB1 and HIF1A, were highly expressed at the end stage (PT‐C2). GO analysis indicated their functions were primarily enriched in responses to hypoxia, endoplasmic reticulum stress, inflammatory responses, cytokine production, leukocyte activation and myeloid cell differentiation. Genes in Cluster 3 tended to be downregulated during pseudotime, with GO enrichment analysis revealing their involvement in kidney development processes, such as urogenital system development (Figure 2I). To further investigate the transition states of proximal tubule cells in pre‐ and post‐IRI samples, we separately analysed the trajectories of these cells. PT‐C2 was primarily located at the terminal ends in pseudotime trajectories (Figure 2E). We next investigated the injury changes associated with transitional states. Injury, ER stress and inflammation‐related genes, including VCAM1, CCL20, VIM, S100A2, HSPA5 and TNFRSF12A, tended to be upregulated in pseudotime in proximal tubule cells in both pre‐and post‐IRI samples, with higher expression in post‐IRI samples (Figure 2H). In summary, in post‐IRI samples, PT‐C2 became progressively dysfunctional, increasing hypoxia, inflammation and apoptosis during pseudotime trajectories. This comprehensive analysis underscores the pivotal role of specific gene expressions in progressing towards a dysfunctional state in proximal tubule cells following injury, highlighting potential targets for therapeutic intervention in kidney repair processes.

**FIGURE 2:**
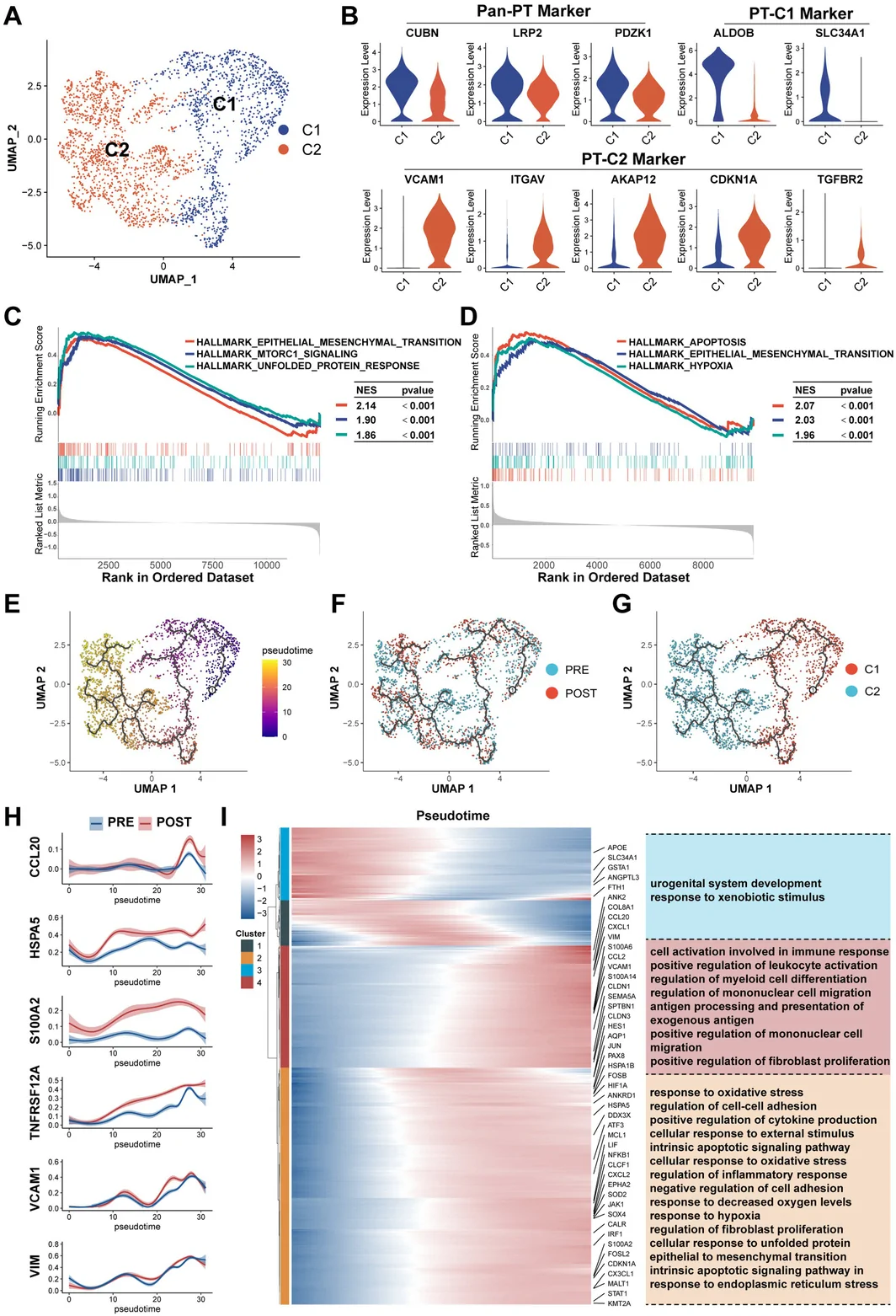
Annotation and Cell States of Proximal Tubule Subsets pre‐ and post‐IRI. (A and B) UMAP plot (A) of PT cell clusters identified based on the expression of highly variable genes and violin plots (B) showing proximal tubule marker expression in the two subclusters. (C and D) The top 3 differentially enriched pathways were shown by GSEA in PT‐C1 (C) and PT‐C2 (D) between pre‐ and post‐IRI. (E) PTC pseudotime analysis. (F and G) Trajectory analysis shows the dynamic process of the proximal tubule cells. RNA expression profiles change continuously along the anatomic axis in pre/post‐IRI (F) and PT subclusters (G). (H and I) Two‐dimensional plots (H) showing the dynamic expression of inflammatory and injury genes during the proximal tubule cell transitions along the pseudotime in pre‐ (blue) and post‐ (red) IRI. Heatmap (I) showing the dynamic changes in gene expression along the pseudotime.

### 3.4 Overview of Ischemia–Reperfusion Injury Effects on Other Cell Types

In the early stage of RIRI, endothelial cells exhibited a marked activation of stress pathways such as the unfolded protein response, TNF/NF‐κB pathway, hypoxia and apoptosis (Figure S3). In the loop of Henle ascending limb (LOH (AL)), genes involved in ion transport functions, including KCNJ1, KCNJ10, CASR and WNK4, were significantly downregulated post‐IRI, suggesting increased hypoxia, apoptosis and ion transport dysfunction (Figure S4). Collecting duct cells showed high expression of NGAL, a marker for AKI and exhibited stress responses such as oxidative stress, hypoxia and the EMT (Figure S5). Stromal cells, including fibroblasts, myofibroblasts and pericytes, demonstrated increased expression of genes related to hypoxia, fibrosis and inflammation. Functional enrichment analysis revealed their involvement in mesenchymal cell differentiation, extracellular matrix organization and response to transforming growth factor beta (TGF‐β) (Figure S6). Collectively, these findings underscore that ischemia–reperfusion injury elicits complex, cell type–specific stress responses that extend beyond tubular epithelial injury. The interplay between endothelial activation, tubular dysfunction and stromal fibrogenesis delineates early pathogenic networks that may serve as therapeutic targets to attenuate injury progression and facilitate renal repair.

### 3.5 Activation of Epithelial–Mesenchymal Transition in Epithelial Cells After IRI

Gene expression analysis of epithelial cells (Table S5) and GSEA analysis of various epithelial cell differential genes demonstrated significant enrichment of the EMT process, as well as inflammation, hypoxic stress and apoptosis (Figure 3A, Table S5). Furthermore, the analysis of bulk RNA sequencing results indicated that the EMT process was activated after IRI (Figure 3B, Table S6). Immunofluorescence analysis of EMT markers, including α‐catenin, N‐cadherin, vimentin and fibronectin, revealed that α‐catenin expression decreased after IRI, while N‐cadherin, vimentin and fibronectin expression increased (Figure 3C). These findings suggest the EMT process is activated in epithelial cells after IRI.

**FIGURE 3:**
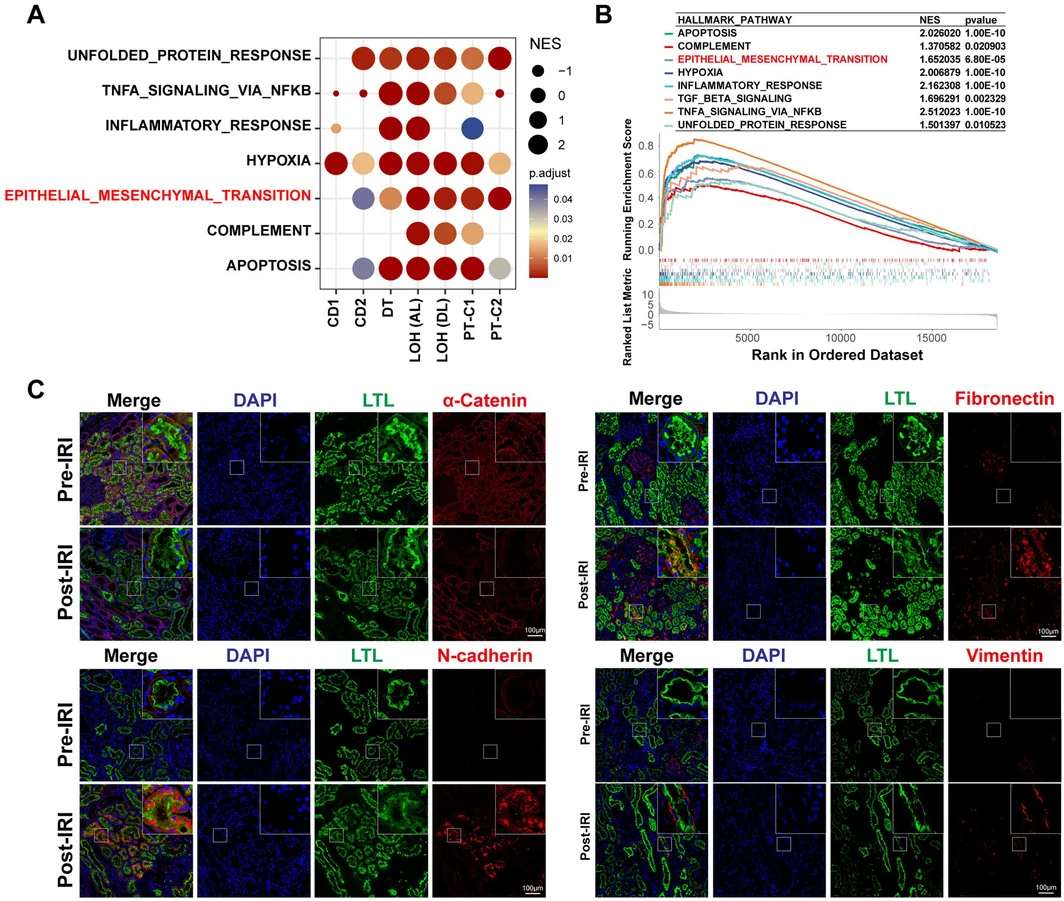
Epithelial‐mesenchymal Transformation of Multiple Renal Tubular Epithelial Cells after IRI. (A) Dot plot shows differentially enriched pathways by GSEA in multiple renal tubular epithelial cells between pre‐ and post‐IRI. (B) Differentially enriched pathways including EMT were revealed by GSEA in bulk‐RNA sequencing between pre‐ and post‐IRI. (C) Representative α‐Catenin, Fibronectin, N‐Cadherin and Vimentin immunofluorescence micrographs of renal tubular epithelial cells on the kidney sections between pre‐ and post‐IRI. Nuclei were counterstained with DAPI (blue). Scale bar = 100 μm.

### 3.6 CD11c ^+^ AREG ^+^ Dendritic Cells Involved in the Promotion of EMT After IRI

Myeloid cell populations were identified using unsupervised clustering, including MC1 (macrophages) and MC2 (renal‐resident DCs) (Figure 4A,B, Figure S7). Notably, the proportion of the MC2 cluster increased after IRI (Figure 4C). DCs, acting as a bridge to mediate the immune response, play a crucial role in mediating IRI [17, 18]. Ligand‐receptor analysis showed that among the myeloid cell clusters, MC2 had a stronger interaction with epithelial cells after IRI through the EGFR‐signalling pathway (Figure 4D,E). The MC2 cluster expressed high levels of AREG and EREG, which are known ligands for EGFR (Figure 4F, Figure S8). Consistent with our human data, both our own mouse single‐cell dataset and publicly available transcriptomic datasets revealed an early and robust upregulation of Areg expression in renal DCs during the initial phase of IRI (Figure S9).

**FIGURE 4:**
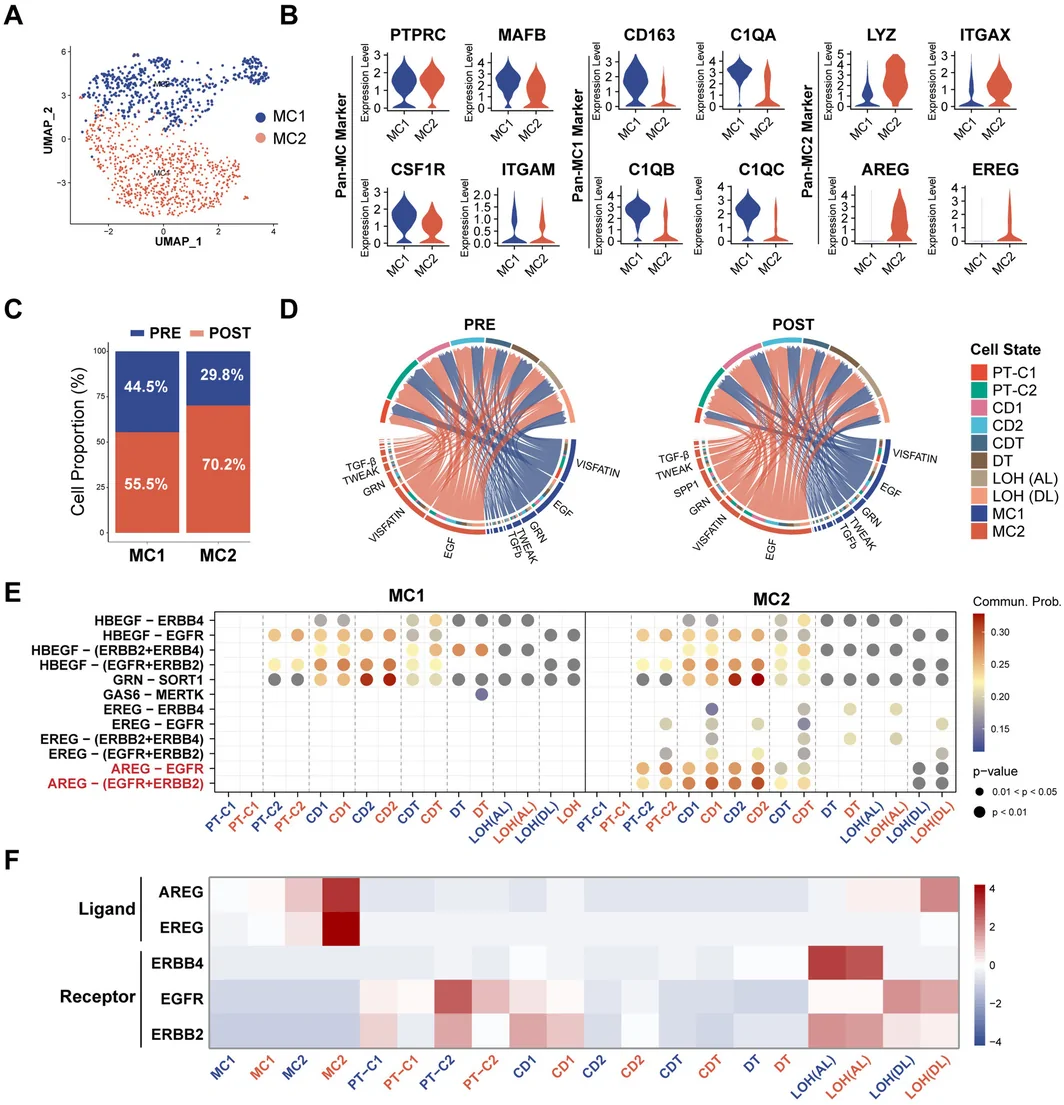
Enhanced communication between CD11c^+^ myeloid cells and epithelial cells. (A and B) UMAP plot (A) of myeloid cell clusters identified based on the expression of highly variable genes and violin plots (B) showing endothelial cell marker expression in the two subclusters. (C) Boxplot shows the percentage of cell types in different groups. (D) The chord diagrams illustrating the top significant signalling pathways from MC1 and MC2 to epithelial cells across pre‐ and post‐IRI conditions. (E) Bubble plot showing the selected ligand‐receptor interactions between myeloid cell subset (MC2) and all renal tubular epithelial cells pre‐ (blue) and post‐IRI (red). *p*‐values are indicated by circle size, with the scale to the right. (F) Heatmap showing the expression of genes in the AREG/EREG‐EGFR signalling pathway in myeloid cells and renal tubular cells pre‐ (blue) and post‐IRI (red).

Immunofluorescence staining revealed high expression of AREG in CD11c^+^ DCs after IRI (Figure 5A). Flow cytometry results confirmed that AREG expression in CD11c^+^ DCs was higher after IRI (Figure 5B, Figure S8A–C). Importantly, AREG expression levels correlated with the severity of kidney injury (*R* = 0.50, *p* = 0.02) and renal tubular apoptosis (*R* = 0.49, *p* = 0.04) (Figure 5C). In a time‐course unilateral ischemia–reperfusion injury(uIRI) model (ischemia for 30 min followed by reperfusion for 1, 3, 6, 12 and 24 h, respectively), Areg expression in the kidney was markedly increased at 1 h of reperfusion, predominantly localized in CD11c^+^ DCs, whereas other immune or parenchymal cell types exhibited minimal expression (Figure S10). These results further validate the existence of this AREG^+^ DCs population and demonstrate that it is activated earlier than other renal cell types, serving as the initial source of Areg expression following ischemia–reperfusion. We next examined the spatial relationship between AREG^+^ DCs and partial EMT in human kidneys. Multiplex immunofluorescence revealed that CD11c^+^AREG^+^ DCs were enriched post‐IRI and positively correlated with partial EMT area (Figure S8D–G), directly linking AREG^+^ DCs to epithelial‐mesenchymal transition in human IRI.

**FIGURE 5:**
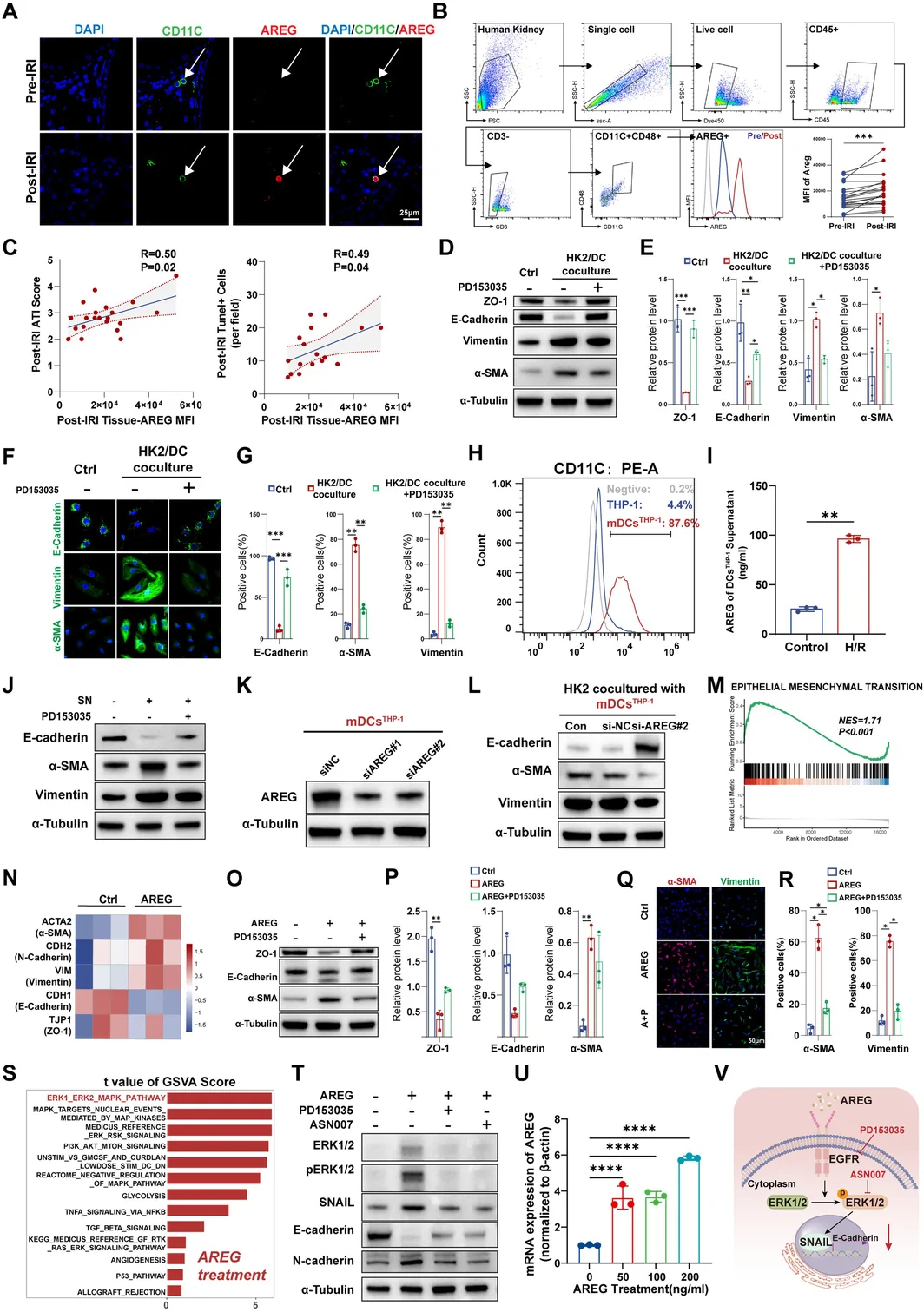
CD11c^+^AREG^+^ DCs are involved in promoting EMT after IRI via the AREG‐EGFR pathway. (A) Representative CD11C (green) and AREG (red) immunofluorescence micrographs of renal myeloid cells on the kidney sections between pre‐ and post‐IRI. Nuclei were counterstained with DAPI (blue). Scale bar = 25 μm. (B) Flow cytometry and paired scatter plot showing the mean fluorescence intensity (MFI) of AREG in CD11c^+^AREG^+^ DCs between pre‐ and post‐IRI (*n* = 21). (C) Correlation of the MFI of AREG in CD11c^+^AREG^+^ DCs with the ATI score (*n* = 21) and the Tunel^+^ cells (*n* = 17) in the tissue after IRI. (D) Western blot showing the ZO‐1 and E‐Cadherin (epithelial markers), α‐SMA and Vimentin (mesenchymal markers) expression at the protein level of HK‐2 cells alone, co‐cultured with CD11c^+^DCs and treatment with PD153035 (EGFR inhibitor) from kidney after IRI. (E) The bar chart showing the relative comparison of ZO‐1, E‐Cadherin, α‐SMA and Vimentin expression (normalized to α‐Tubulin) in all groups (*n* = 3 per group). (F) Representative E‐Cadherin (epithelial markers), α‐SMA and Vimentin (mesenchymal markers) immunofluorescent micrographs of HK‐2 cells alone, co‐cultured with CD11c^+^DCs and treatment with PD153035 from kidney after IRI. Nuclei were counterstained with DAPI (blue). Scale bar = 20 μm. (G) The bar charts showing the percentages of E‐Cadherin, α‐SMA and Vimentin positive in HK2 cells in all groups (*n* = 3 per group). (H) For differentiation into mDCs, 100 ng/mL rhGM‐CSF, 200 ng/mL rhIL‐4, 20 ng/mL TNF‐α and 200 ng/mL ionomycin were added. Flow cytometry analysis comparing the percentage of CD11c^+^ cells before and after induction. (I) Bar chart showing the AREG levels in the supernatant of mDC^THP‐1^ cells after hypoxia/reoxygenation. (J) Western blot showing the protein expression of E‐Cadherin (epithelial marker), α‐SMA and Vimentin (mesenchymal markers) in HK‐2 cells under different conditions: Alone, treated with the supernatant of mDC^THP‐1^ cells after hypoxia/reoxygenation and treated with PD153035 (EGFR inhibitor). (K) Western blot analysis showing the knockdown of AREG in mDC^THP‐1^ cells using AREG siRNA. (L) Western blot showing the protein expression of E‐Cadherin (epithelial marker), α‐SMA and Vimentin (mesenchymal markers) in HK‐2 cells under different conditions: Alone, co‐cultured with mDC^THP‐1^ cells after hypoxia/reoxygenation or AREG knockdown mDC^THP‐1^ cells after hypoxia/reoxygenation. (M) The EMT pathway was enriched by GSEA in HK2 cells between control and AREG treatment. (N) Heatmap shows the expression of ACTA2 (a‐SMA), CDH2 (N‐Cadherin), VIM (Vimentin), CDH1 (N‐Cadherin) and TJP1 (Z0‐1) in HK2 cells between control and AREG treatment. (O) Western blot analysis of ZO‐1, E‐Cadherin and α‐SMA of HK‐2 cells under normoxic conditions at the 48th hour in control, AREG‐treated, AREG and PD153035‐treated groups. (P) The bar chart showing the relative comparison of ZO‐1, E‐Cadherin and α‐SMA expression (normalized to α‐Tubulin) in all groups (*n* = 3 per group). (Q) Representative α‐SMA (red) and Vimentin (green) immunofluorescence micrographs of HK‐2 cells under normoxic conditions at the 48th hour in control, AREG‐treated and AREG and PD153035‐treated groups. (R) The bar charts show the percentages of α‐SMA and Vimentin positive in HK2 cells in all groups (*n* = 3 per group). (S) GSVA enrichment analysis indicated a significant enrichment of ERK1/2‐associated pathways in HK‐2 cells following AREG treatment compared to the control group. (T) Western blot showing the protein expression of ERK1/2, p‐ERK1/2, SNAIL, E‐Cadherin and N‐Cadherin in HK‐2 cells under different conditions: Alone, AREG treatment, AREG + PD153035 (EGFR inhibitor) and AREG + ASN007 (ERK inhibitor). (U) Recombinant AREG induces dose‐dependent upregulation of AREG mRNA in HK‐2 cells. (V) Schematic illustration of the AREG‐induced EMT pathways in HK‐2 cells, highlighting the EGFR‐ERK1/2‐SNAIL signalling axis. *p < 0.05; **p < 0.01; ***p < 0.001; ****p < 0.0001.

In HK‐2 cells co‐cultured with CD11c^+^ DCs from human, E‐cadherin and the epithelial junction protein ZO‐1 were downregulated, while vimentin and α‐SMA were upregulated. These effects were significantly mitigated by the EGFR inhibitor PD153035, indicating that AREG^+^ DCs promote EMT in HK‐2 cells through the EGFR signalling pathway (Figure 5D–G). THP‐1 cells are commonly used to study the function of myeloid cells [29]. We differentiated THP‐1 cells into mDCs^THP‐1^ cells and confirmed successful differentiation by detecting high CD11C expression through flow cytometry (Figure 5H). We exposed differentiated mDCs^THP‐1^ cells to 12 h of hypoxia followed by 6 h of reoxygenation, resulting in a significant increase in AREG levels in the supernatant (Figure 5I). Moreover, this supernatant triggered EMT in HK‐2 cells but was inhibited by PD153035 (Figure 5J). To further validate the role of AREG, we silenced its expression in mDCs^THP‐1^ cells using siRNA, followed by hypoxia/reoxygenation (H/R) and co‐culture with HK‐2 cells. Strikingly, AREG knockdown significantly reduced the EMT‐promoting effect on HK‐2 cells (Figure 5K,L).

Bulk RNA sequencing of HK‐2 cells treated with 100 ng/mL AREG recombinant protein showed significant enrichment of EMT signals, with decreased expression of E‐cadherin and ZO‐1 and increased expression of vimentin, N‐cadherin and α‐SMA (Figure 5M,N, Table S7). Furthermore, EMT activation was also observed in HK‐2 cells treated with 100 ng/mL recombinant AREG protein, a process significantly inhibited by PD153035 (Figure 5O–R). GSVA enrichment analysis revealed a significant enrichment of ERK1/2‐associated pathways in HK‐2 cells after AREG treatment compared to the control group (Figure 5S, Table S8). AREG recombinant protein treatment enhanced ERK phosphorylation and upregulated SNAIL, a key repressor of E‐cadherin, leading to E‐cadherin downregulation and EMT induction. This effect was mitigated by the EGFR inhibitor PD153035 or the ERK inhibitor ASN007 (Figure 5T). Additionally, AREG treatment induced a positive feedback loop [30, 31, 32], increasing AREG expression in HK‐2 cells, potentially via the EGFR–ERK pathway (Figure 5U).

These findings suggest that CD11c^+^ DCs upregulate AREG after IRI, driving epithelial EMT via the AREG‐EGFR‐ERK‐SNAIL pathway (Figure 5V).

### 3.7 AREG Knockdown or Depleting CD11c ^+^ DCs Mitigates Renal Injury and Fibrosis Following IRI

To further investigate the role of AREG in AKI‐CKD transition, we utilized a shRNA‐mediated strategy to modulate AREG expression in vivo in the kidney [33]. We employed a hydrodynamic‐based gene delivery approach to intravenously administer a plasmid vector encoding an Areg‐targeting interfering sequence (PLKO.1‐shAreg) into mice. Two days after injection, the mice underwent uIRI surgery and were analysed at 2 h, 1 day and 14 days post‐surgery (Figure 6A). Western blot analysis confirmed the efficient knockdown of Areg expression (Figure 6B). Immunofluorescence staining further revealed that Areg expression in CD11c^+^ DCs increased at 2 h, peaked at 1 day and declined by 14 days following IRI, whereas Areg expression was scarcely detectable in the knockdown group (Figure 6C,D). Following RIRI, acute tubular injury and subsequent fibrosis were markedly exacerbated, as evidenced by the loss of tubular brush borders and epithelial cells, increased tubular apoptosis and extensive fibrotic lesions in the distal phase. In contrast, Areg knockdown significantly alleviated these pathological alterations and promoted renal recovery, which was accompanied by a pronounced reduction in fibrosis‐related proteins, including α‐SMA and collagen I, particularly during the late fibrotic stage (Figure 6E–H, Figure S11A,B). To further investigate whether distal renal fibrosis is associated with EMT, we assessed the expression of heat shock protein 47 (Hsp47), a collagen synthesis–related protein and vimentin, an EMT marker. Both proteins progressively accumulated during reperfusion, whereas their expression was markedly reduced in Areg knockdown mice (Figure 6I,J, Figure S11C,D). Collectively, these findings demonstrate that Areg knockdown attenuates RIRI in mice, particularly by suppressing distal fibrosis through inhibition of EMT‐related pathways.

**FIGURE 6:**
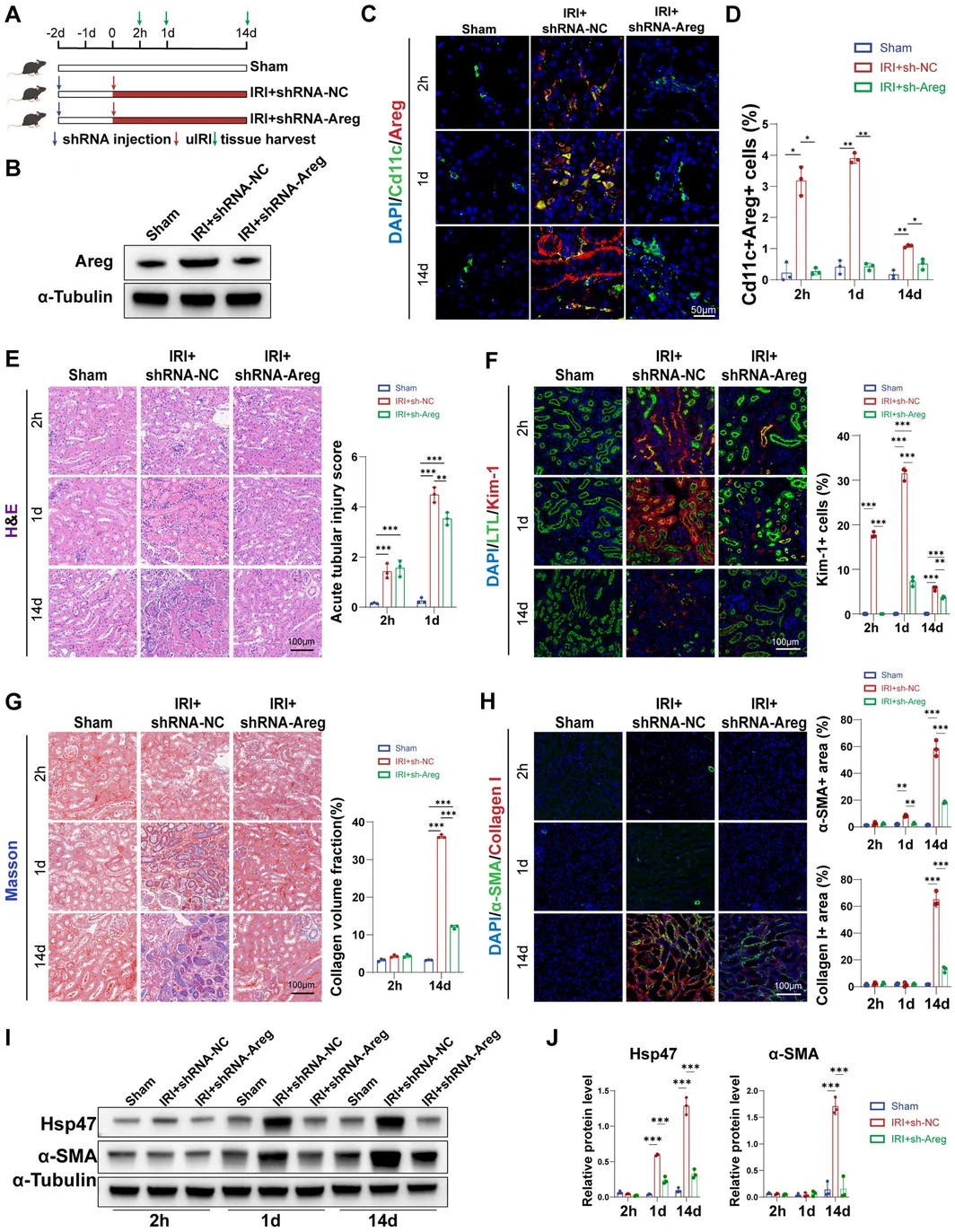
Areg knockdown alleviates renal injury and fibrosis following RIRI in mice. (A) Experimental design. Blue arrows indicate sh‐Areg plasmid injection. Red arrows indicate the time of IRI surgery. Green arrows indicate the time of tissue harvest. (B) Representative Western blot shows renal Areg protein expression in different groups as indicated. Bar chart shows the relative Areg protein levels in different groups (*n* = 3 per group). (C) Representative Cd11c (green) and Areg (red) immunofluorescent micrographs of renal myeloid cells on the kidney sections of uIRI mouse model in all groups. Nuclei were counterstained with DAPI (blue). Scale bar = 50 μm. (D) The bar charts show the percent of Cd11c^+^Areg^+^ cells of uIRI mouse model in all groups (*n* = 3 per group). (E) Representative micrographs show kidney injury in different groups as indicated. Images of H&E staining were shown. Scale bar = 100 μm. The bar charts show the ATI score in all groups. (F) Representative Kim‐1 (red) and LTL (green) immunofluorescent micrographs on the kidney sections of uIRI mouse model in all groups. Nuclei were counterstained with DAPI (blue). Scale bar = 100 μm. The bar charts show the percentage of Kim‐1+ cells in all groups. (G) Representative micrographs show kidney fibrosis in different groups as indicated. Images of Masson's trichrome staining were shown. Scale bar = 100 μm. The bar charts show the collagen volume fraction in all groups. (H) Representative α‐SMA (green) and Collagen I (red) immunofluorescent micrographs of the kidney sections of uIRI mouse model in all groups. Nuclei were counterstained with DAPI (blue). Scale bar = 100 μm. The bar charts show the percent of α‐SMA^+^ area and Collagen I^+^ area of uIRI mouse model in all groups, respectively. (I and J) Representative Western blot shows renal Hsp47, Vimentin and α‐SMA protein expression in different groups as indicated. Bar chart shows the relative Hsp47, Vimentin and α‐SMA protein levels in different groups (*n* = 3 per group). *p < 0.05; **p < 0.01; ***p < 0.001.

To further confirm the pathogenic role of DCs as the predominant source of AREG in the kidney, we employed CD11c‐DTR transgenic mice to selectively ablate CD11c^+^ DCs via diphtheria toxin (DTX) administration (Figure 7A). Flow cytometric analysis verified the efficient depletion of renal CD11c^+^ cells, confirming successful establishment of the DC‐depleted model (Figure 7B,C). Immunofluorescence staining further revealed markedly reduced AREG expression in kidney tissues following DC ablation, indicating that CD11c^+^ DCs are the predominant source or trigger of AREG during IRI (Figure 7D).

**FIGURE 7:**
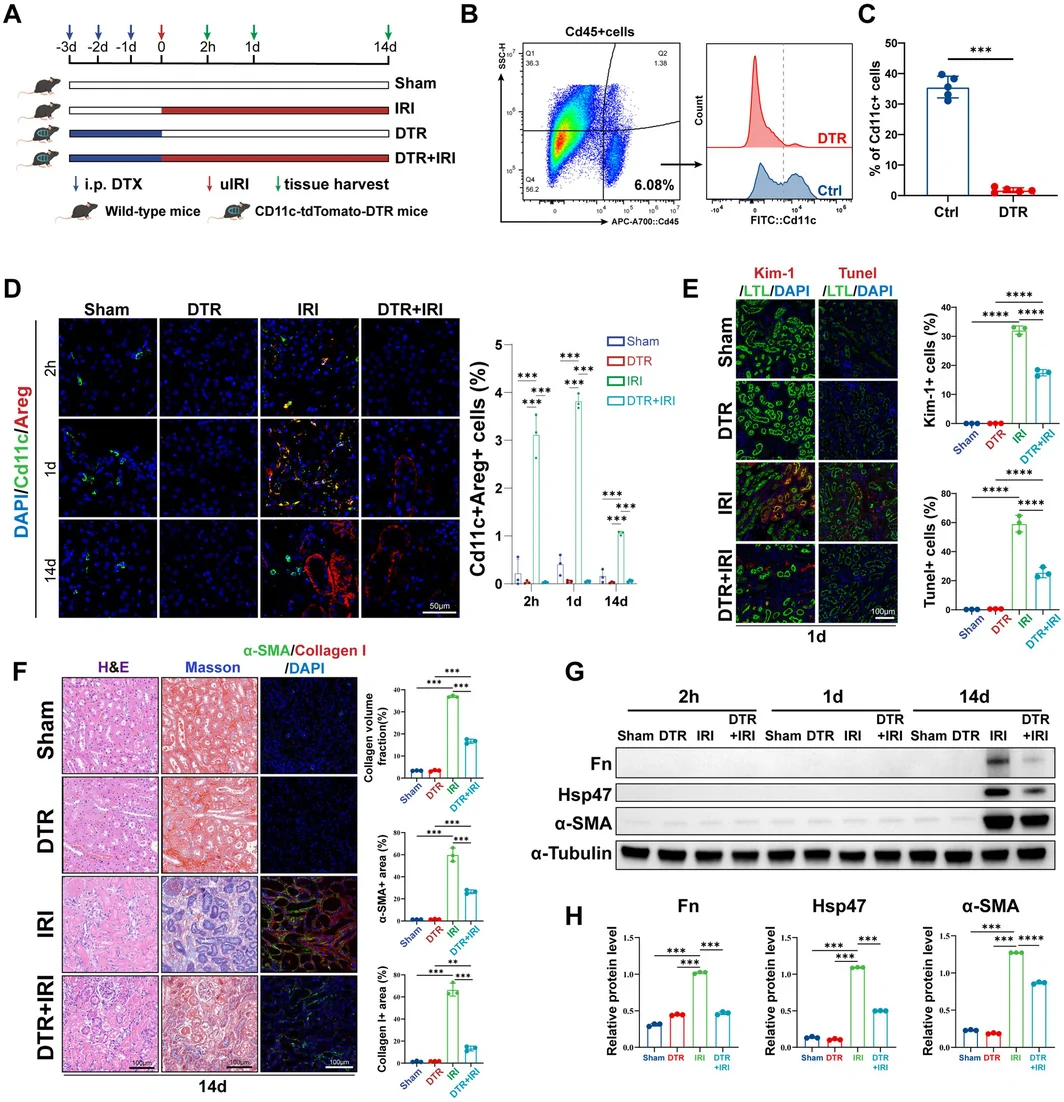
Depletion of CD11c^+^ DCs alleviates renal injury and fibrosis following RIRI in mice. (A) Schematic illustration of the experimental design. Wild‐type and CD11c‐tdTomato‐DTR transgenic mice were intraperitoneally injected with diphtheria toxin (DTX) 3 days before surgery, followed by unilateral ischemia–reperfusion injury (uIRI) and kidney tissue collection at 2 h, 1 day and 14 days post‐injury. B: Representative flow cytometry plots showing depletion efficiency of CD11c^+^ cells in DTR mice after DTX injection. (C) Quantification of CD11c^+^ cells among CD45^+^ leukocytes in control and DTR mice. (D) Representative immunofluorescent staining showing co‐expression of Cd11c (green) and Areg (red) in renal myeloid cells in kidney sections from all groups at 2 h, 1 day and 14 days after uIRI. Nuclei were counterstained with DAPI (blue). Scale bar = 50 μm. The bar chart shows the percentage of Cd11c^+^Areg^+^ cells in each group (*n* = 3). (E) Representative Kim‐1 (red) or TUNEL assay (red) and LTL (green) immunofluorescent micrographs on the kidney sections of uIRI mouse model in all groups. Nuclei were counterstained with DAPI (blue). Scale bar = 100 μm. The bar charts show the percentage of Kim‐1+ cells and percentage of Tunel^+^ cells in all groups (*n* = 3). (F) Representative micrographs show kidney fibrosis in different groups as indicated. Images of H&E staining, Masson's trichrome staining and representative α‐SMA (green) and Collagen I (red) immunofluorescence micrographs were shown. Scale bar = 100 μm. The bar charts show the collagen volume fraction, percent of α‐SMA^+^ area and Collagen I^+^ area in all groups (*n* = 3). (G and H) Representative Western blot (G) showing expression levels of fibronectin (Fn), heat shock protein 47 (Hsp47) and α‐smooth muscle actin (α‐SMA) in the kidney from different groups as indicated. The bar chart (H) shows relative protein levels of Fn, Hsp47 and α‐SMA normalized to α‐tubulin (*n* = 3). *p < 0.05; **p < 0.01; ***p < 0.001; ****p < 0.00.

Functionally, DC depletion markedly alleviated renal tubular injury and apoptosis, as demonstrated by decreased Kim‐1 expression and reduced TUNEL^+^ cells (Figure 7E). Histological evaluation using H&E and Masson's trichrome staining showed improved renal morphology and reduced collagen deposition in DTX‐treated DTR mice compared with controls (Figure 7F). Consistently, both immunofluorescence staining and Western blot analysis demonstrated a pronounced reduction in fibrosis‐related markers, including α‐SMA, collagen I, fibronectin (Fn) and Hsp47 in DC‐depleted kidneys (Figure 7F–H).

Collectively, these findings demonstrate that CD11c^+^ DCs serve as the principal source of AREG in the kidney and that their ablation significantly mitigates RIRI–induced tubular injury and interstitial fibrosis. However, whether the protective effect of DC ablation is specifically attributable to AREG derived from DCs, rather than from other cellular sources, remains to be determined.

### 3.8 AREG‐Deficient DCs Fail to Induce Fibrosis Upon Adoptive Transfer Into DC‐Depleted Mice

To directly test whether DC‐derived AREG mediates renal fibrosis, we adoptively transferred BMDCs transfected with control siRNA (siNC) or Areg‐specific siRNA (siAreg) into CD11c‐DTR mice after DT‐mediated DC ablation, followed by uIRI. Recipients of siNC BMDCs developed significant interstitial fibrosis at day 14, comparable to non‐depleted controls (DTR‐negative littermates receiving PBS). In contrast, recipients of siAreg BMDCs remained largely protected, with significantly attenuated fibrosis as assessed by Masson trichrome, Sirius Red, α‐SMA immunohistochemistry and collagen I staining (Figure S12). These results demonstrate that AREG expression in DCs is necessary for the profibrotic effect of DCs following IRI.

## 4 Discussion

IRI is a critical factor in AKI and presents significant risks to human health, often leading to conditions such as AKI in severe cases [2]. Currently, AKI is primarily managed with supportive care, but outcomes remain poor [34]. Treatments that have shown effectiveness in animal studies, such as ANP and IGF‐1, often fail to demonstrate similar efficacy in clinical settings [9, 10]. This highlights the urgent need for a human IRI model, which aims to better understand the underlying mechanisms and identify new therapeutic targets for treatment. Previous studies have shown that temporary clamping and relaxing of the renal artery during PN impairs short‐ and long‐term renal function [13, 14]. Thus, kidneys affected by IRI during PN serve as valuable specimens for studying human renal IRI. However, the obtainment of normal renal tissue before and after PN poses risks, including bleeding [16].

To circumvent these risks, we simulated the IRI scenario in RN patients and obtained tissues for scRNA‐seq analysis. We discovered that in the early stages of human RIRI, there is significant activation of inflammatory responses and EMT pathway in epithelial cells and revealed enhanced interactions between CD11c^+^AREG^+^ DCs and epithelial cells post‐RIRI. Additionally, Areg knockdown and DCs deletion in the mouse uIRI model significantly reduced acute tubular damage, apoptosis and long‐term fibrosis.

Accumulating evidence indicates that single‐cell transcriptome analysis is a breakthrough method for studying the pathogenesis of AKI. Recent scRNA‐seq studies on the mouse IRI model have analysed the mononuclear phagocytic system, demonstrating the complementary roles of kidney‐resident macrophages and monocyte‐derived infiltrating macrophages in regulating inflammation and promoting tissue repair [35]. These studies identified macrophage subsets crucial to AKI pathogenesis, revealing the complexity and heterogeneity of AKI. However, they never studied intercellular communication between immune cells and parenchymal cells and, most importantly, did not use human specimens. This study conducted single‐cell sequencing of human kidneys undergoing IRI and comprehensively characterized the transcriptional changes in the human kidney before and after IRI. We characterized the cellular composition of adult kidneys, comparing post‐IRI to pre‐IRI samples. Interestingly, PT‐C2 cluster gene expressions were associated with the failure of renal tubular repair, similar to findings in mice with RIRI [28]. Failures in this repair process may result in EMT, as illustrated in Figure 4. EMT plays a significant role in the progression of renal fibrosis. Our study using the human RIRI model observed that cells failing to adequately repair during the early stages of ischemia–reperfusion underwent EMT. This phenomenon could partially explain our previous study [12], which showed the long‐term development of fibrosis in kidneys following partial nephrectomy (Figure 1A).

The large number of patients and captured cells allowed for a global characterization of the IRI renal microenvironment and cell–cell communication. This comprehensive analysis enabled the identification of rare cell subsets, such as renal MC2, in patients with AKI. Flow cytometry analysis and multiplex immunohistochemistry (mIHC) of kidney sections consistently supported the conclusion that the MC2 population increased significantly in most post‐IRI samples, albeit with varying amounts. Further, we proposed a role for MC2‐mediated inflammatory networks in the pathogenesis of RIRI and subsequent failure of renal repair leading to fibrosis. CD11c^+^ DCs highly express AREG post‐IRI and promote EMT in epithelial cells via the AREG‐EGFR pathway. This finding aligns with recent studies indicating that proximal tubular epithelial cells (PTECs) can promote kidney inflammation by interacting with dendritic cells and that specific tubulointerstitial inflammatory states are related to progressive kidney diseases [36, 37]. These conclusions may provide new insights into the pathogenesis of human RIRI and open new avenues for therapeutic exploitation.

Immune dysfunction is a prominent feature of AKI, with DCs playing a key role in coordinating innate and adaptive immune responses, including the induction of protective proinflammatory and tolerogenic immune responses [38]. DCs can be categorized into different subsets, each exhibiting significant phenotypic heterogeneity and functional plasticity [39]. A recent large‐scale scRNA‐seq study identified a rare population of DCs characterized by CD11c^+^CD163^+^ expression, playing an important role in impaired proximal tubular epithelial cell (iPTEC)‐DC3‐T cell interactions in lupus nephritis progression [36]. Using scRNA‐seq, flow cytometry and mIHC staining, we identified the kidney MC2 population in RIRI patients, characterized by CD11c^+^AREG^+^ expression. Ligand‐receptor analysis showed that the MC2 subpopulation had enhanced communication with iPTECs through the AREG‐EGFR pathway, leading to an obvious EMT phenotype in iPTECs. Previous studies have reported that resident DCs are the major ‘first responders’ for secreting TNF and other inflammatory mediators involved in the early pathophysiological events leading to kidney dysfunction in RIRI [17]. During the transition from normal renal repair to maladaptive fibrosis in RIRI, sustained GM‐CSF expression in tubular cells significantly activates DCs and induces T cells to secrete proinflammatory cytokines TNF‐α and IL‐1β [40]. We speculated that CD11c^+^ DCs highly express AREG post‐IRI and promote EMT in epithelial cells via the AREG‐EGFR pathway. In addition, a DC deletion murine model was used to validate DCs play a crucial role in RIRI and kidney fibrosis in the present study.

Recently, AREG has been identified as an important factor in myofibroblast proliferation in various diseases and AREG, in combination with EGFR, amplifies renal fibrosis in the proximal tubule [41]. Inhibition of AREG is an attractive therapeutic strategy for fibrotic diseases. Previous studies have reported that silencing Areg in vivo can inhibit the progression of renal fibrosis in unilateral ureteral obstruction (UUO) mice by blocking the EGFR pathway [42]. The EGFR signalling pathway is also involved in SMAD2/3‐mediated TGF‐β1‐induced fibrotic diseases [43]. However, none of these studies identified the source of Areg in RIRI. Our study showed that the DC subset MC2, which appears early after IRI, highly expresses Areg and induces EMT in renal tubules through paracrine action on the EGFR pathway. Blocking Areg reduced EMT in renal tubules and improved renal tissue damage in the uIRI‐AKI mouse model, ultimately mitigating renal fibrosis at the later stage. These data suggest that inhibiting Areg can be used as a precise intervention strategy to reduce inflammatory tissue damage in the early stages of AKI.

In further clinical sample detection, we conducted a retrospective correlation study using kidney specimens from RIRI patients. We found that the level of renal Areg in RIRI patients was significantly increased and its expression in DC subpopulations MC2 was positively correlated with the ATI score and the degree of tubular cell apoptosis post‐IRI. Previous studies have reported that IRI‐induced AKI is a major contributor to renal fibrosis and CKD [34, 44]. In a previous study, we analysed patients who underwent clamped PN followed by RN for tumour recurrence. IRI in their kidneys provided insight into clamped PN effects. Histological analysis showed worsened CKD scores and increased fibrosis in RN samples compared to PN samples [12]. Moreover, our other studies demonstrated that the severity of AKI during PN is strongly correlated with functional decline and parenchymal atrophy in the operated kidney, further confirming the long‐term impact of IRI‐induced AKI on the surgical kidney [13, 14]. In our current study, we found that DCs promote EMT in renal tubular epithelial cells, potentially acting as a key initiating factor in the progressive morphological and functional deterioration of the kidney. These data support a potential pathogenic role of CD11c^+^AREG^+^ DCs in human AKI, suggesting that AREG may become a new therapeutic target.

Our study employed RN to simulate IRI in renal tissues, but several limitations should be noted. The reperfusion time was constrained to 28 min (median) due to the standard duration of the surgical procedure, potentially restricting the portrayal of renal injury mechanisms that typically manifest over more extended reperfusion periods observed in clinical settings. Moreover, the complete removal of the kidney during the RN procedure presented challenges in obtaining renal tissue samples at various time points post‐reperfusion, thereby limiting our capacity to examine the dynamic changes and progression of renal injury over time. Furthermore, the excision of the diseased kidney prevented the assessment of alterations in serum creatinine or urine biomarkers in the same patients, impeding our ability to assess systemic biomarkers as indicators of renal function and injury following IRI. Additionally, although this is, to our knowledge, the first human RIRI model established using radical nephrectomy specimens—providing unique insights into ischemia‐induced EMT at single‐cell level—it represents surgical clamping‐induced warm ischemia and may not fully recapitulate RIRI from systemic causes (e.g., sepsis, shock). Extrapolation to other RIRI subtypes therefore requires caution and further investigation. Despite these constraints, our model offers valuable insights into the immediate effects of IRI. Future studies could benefit from incorporating alternative models or supplementary methodologies to capture the temporal aspects and systemic effects of RIRI more comprehensively.

This study utilized a human RIRI model to comprehensively characterize early single‐cell transcriptomic changes in the human kidney following ischemia–reperfusion injury, offering valuable insights into the mechanisms underlying ischemia–reperfusion‐induced renal damage. CD11c^+^AREG^+^ DCs initiate and promote EMT in renal tubular epithelial cells via the AREG‐EGFR signalling pathway, which highlights these cells as potential therapeutic targets for mitigating RIRI.

## Author Contributions

**Xin Luo:** conceptualization, methodology, software, validation, formal analysis, data curation, visualization, project administration, writing – original draft. **Longbin Xiong:** methodology, software, validation, formal analysis, investigation, data curation, writing – original draft. **Zhaohui Zhou:** methodology, software, validation, investigation, data curation. **Lihao Zhang:** validation, investigation, data curation. **Yulu Peng:** methodology, software, data curation, formal analysis. **Xiangpeng Zou:** investigation, resources, data curation. **Yixin Huang:** investigation, resources, data curation. **Yisong Lin:** software, validation, formal analysis, data curation. **Xiaofeng Yang:** resources, investigation, data curation. **Zhanji Zhan:** methodology, resources, investigation. **Fangjian Zhou:** methodology, resources, supervision. **Youhua Liu:** conceptualization, methodology, supervision, writing – review and editing. **Chunping Yu:** validation, supervision, writing – review and editing. **Baokui Ye:** conceptualization, data curation, formal analysis, validation, visualization, supervision, writing – original draft, writing‐review and editing. **Zhiling Zhang:** conceptualization, resources, supervision, project administration, funding acquisition, writing – review and editing. All authors have read and approved the final version of the manuscript.

## Funding

This study was supported by the National Natural Science Foundation of China (No. 82273031, 82273311, 82303922, 82373225, 82472655, 82503619, 82503476 and 82573767), the China Postdoctoral Science Foundation (NO. 2023M734038 and 2024M763768), the Guangdong Natural Science Foundation (No. 2024A1515010303), the Guangzhou Science and Technology Project (No. 2024A04J4319), the Chih Kuang Scholarship for Outstanding Young Physician‐Scientists of Sun Yat‐sen University Cancer Center (CKS‐SYSUCC‐2024006 and CKS‐SYSUCC‐2025008), the Guangzhou Basic Research Program‐City‐University(Hospital)‐Enterprise Joint Funding Project (2025A03J3620) and the Guangzhou Medical Key Subject Construction Project (2025–2027).

## Conflicts of Interest

The authors declare no conflicts of interest.

## Supporting information


**Data S1:** Supporting Information.

**Figure S1:** Correlation between Ischemia Time and AIRD or Parenchymal Volume Change in Patients without Pre‐existing Diseases.
A: Ischemia time was positively correlated with the *acute ipsilateral renal dysfunction (AIRD) score* (*R* = 0.24, *p* = 0.001).
B: Ischemia time was also positively correlated with the percentage decrease in ipsilateral renal parenchymal volume (RPV) (*R* = 0.16, *p* = 0.007).
**Figure S2:** Additional Information on Single‐cell Sequencing.
A: The table shows three pairs of biopsy samples that underwent single‐cell sequencing and their cell counts and survival rates.
B–E: The UMAP plots show celltypes (B), clusters origin (C), groups origin (D) and samples origin (E) by colour.
**Figure S3:** Annotation and Cell States of Endothelial Subsets pre‐ and post‐IRI.
A, B: UMAP plot (A) of endothelial cell clusters identified based on the expression of highly variable genes and violin plots (B) showing endothelial cell marker expression in the three subclusters.
C: Dot plot shows differentially enriched pathways by GSEA in endothelial cell subsets between pre‐ and post‐IRI.
D: PPI network for top 20 hub genes of differentially expressed genes in endothelial cells between pre‐ and post‐IRI.
E: Violin plots showing expression of part of the hub gene in the three subclusters between pre‐ and post‐IRI.
F: Representative ICAM1 immunofluorescence micrographs of renal endothelial cells on the kidney sections between pre‐ and post‐IRI. Nuclei were counterstained with DAPI (blue). Scale bar = 100 μm.
**Figure S4:** Annotation and Cell States of LOH subsets pre‐ and post‐IRI.
A, B: Heatmap (A) shows the expression of marker genes in the LOH (AL) and LOH (DL). UMAP plot (B) represents the subtypes of LOH (AL) and LOH (DL) cells from the pre/post‐IRI sample.
C: Feature plots showing representative gene expression for functional metabolite handling in LOH cells.
D: Violin plots showing representative gene expression for functional metabolite handling in LOH cells between pre‐ and post‐IRI.
E: LOH (AL) pseudotime analysis.
F: Trajectory analysis shows the dynamic process of the LOH (AL) cells. RNA expression profiles change continuously along the anatomic axis in pre/post‐IRI.
G‐H: Two‐dimensional plots (G) showing the dynamic expression of functional metabolite handling genes during the LOH (AL) cell transitions along the pseudotime in pre‐ (blue) and post‐ (red) IRI. Heatmap (H) showing the dynamic changes in gene expression along the pseudotime.
**Figure S5:** Annotation and Sell States of Collecting Duct Subsets pre‐ and post‐IRI.
A, B: UMAP plot (A) of collecting duct cell clusters identified based on the expression of highly variable genes and violin plots (B) showing collecting duct marker expression in the two subclusters.
C: Collecting duct cells pseudotime analysis.
D, E: Trajectory analysis shows the dynamic process of the collecting duct cells. RNA expression profiles change continuously along the anatomic axis in pre/post‐IRI (D) and collecting duct subclusters (E).
F: Trajectory analysis shows RNA expression change of LCN2 along the anatomic axis.
G, H: Two‐dimensional plots (G) showing the dynamic expression of inflammatory and injury genes during the collecting duct cell transitions along the pseudotime in pre‐ (blue) and post‐ (red) IRI. Heatmap (H) showing the dynamic changes in gene expression along the pseudotime.
**Figure S6:** Analyses of Stromal Cells.
A, B: UMAP plot (A) of stromal cell clusters identified based on the expression of highly variable genes and violin plots (B) showing stromal cell marker expression in the three subclusters.
C: Collecting duct cells pseudotime analysis.
D, E: Trajectory analysis shows the dynamic process of the fibroblast and myofibroblast. RNA expression profiles change continuously along the anatomic axis in pre/post‐IRI (D) and the two subclusters (E).
F, G: Two‐dimensional plots (F) showing the dynamic expression of inflammatory and hypoxia genes during the stromal cell transitions along the pseudotime in pre‐ (blue) and post‐ (red) IRI. Heatmap (G) showing the dynamic changes in gene expression along the pseudotime.
**Figure S7:** Marker genes of renal resident dendritic cells and plasmacytoid dendritic cells
**Figure S8:** Identification and characterization of human renal CD11c⁺ dendritic cells.
(A, B) CD11C + AREG+ DCs in Human Dignature Genes.
(C) Representative flow cytometry gating strategy for sorting CD11c⁺ DCs from human renal tissue after RIRI using conventional DC markers (CD45⁺CD11c⁺HLA‐DR⁺CD14⁻).
(D) Representative multiplex immunofluorescence (mIF) images of human kidney biopsies pre‐ and post‐IRI. Sections were stained for E‐cadherin (green), Vimentin (red), CD11c (cyan), AREG (magenta) and DAPI (blue). Scale bar = 50 μm.
(E) Quantification of CD11c⁺AREG⁺ DC density (cells/mm^2^) in pre‐IRI versus post‐IRI kidneys. Data are individual values with mean ± SEM. ***p < 0.001 by paired *t*‐test.
(F) Quantification of partial EMT area (defined as Pan‐CK⁺ tubules with E‐cadherin loss and Vimentin upregulation) as a percentage of total tubular area. *p < 0.05 by paired *t*‐test.
(G) Correlation analysis between partial EMT area (%) and CD11c⁺AREG⁺ DC density (cells/mm^2^). Each dot represents a field of view (*n* = 21). *R* = 0.44, *p* = 0.04.
**Figure S9:** Single‐cell RNA Sequencing Analysis of Renal Dendritic Cell Subsets after uIRI.
A–C: UMAP visualization of single‐cell transcriptomes showing cluster origins (A), group distribution (B, sham vs. uIRI) and annotated cell types (C).
D: Dot plot depicting canonical marker gene expression across major renal cell populations.
E: Dot plot showing expression levels of Itgax and Areg among different cell types, with Areg highly expressed in dendritic cells (DCs).
F: Violin plots showing expression of marker genes used to define dendritic cell subsets (Xcr1, Clec9a, Btla, Cd209a, Itgam, Irf4).
G: Violin plots illustrating Itgax and Areg expression within DC subsets, with Areg predominantly expressed in cDC2 cells, especially elevated after uIRI.
H, I: Validation using external datasets (GSE139107 and GSE180420) confirmed that Areg expression in dendritic cells is markedly upregulated during the early phase of ischemia–reperfusion injury, followed by a decline at later stages.
**Figure S10:** Dynamic Changes in AREG Expression in Renal CD11c⁺ Cells after uIRI.
A: Experimental design illustrating the uIRI model and kidney tissue collection at 1, 3, 6, 12 and 24 h post‐reperfusion.
B: Flow cytometry gating strategy used to identify CD45⁺CD11c⁺AREG⁺ cells from kidney single‐cell suspensions.
C, D: In CD45⁺CD11c⁺ cells, AREG expression was significantly increased as early as 1 h after reperfusion, reaching a peak in the early phase.
E, F: In contrast, AREG upregulation in CD11c⁻ cells was detected later, becoming evident at 12 h after reperfusion, indicating a delayed expression pattern compared with CD11c⁺ cells.
**p < 0.01; ***p < 0.001.
**Figure S11:** Changes in renal Hsp47, Vimentin and α‐SMA expression among groups.
A: Representative Ngal (red) and LTL (green) immunofluorescence micrographs on the kidney sections of uIRI mouse model in all groups. Nuclei were counterstained with DAPI (blue). Scale bar = 100 μm. The bar charts showing the percentage of Ngal+ cells in all groups.
B: representative images of TUNEL assay of on the kidney sections of uIRI mouse model in all groups. Nuclei were counterstained with DAPI (blue). Scale bar = 50 μm. The bar charts showing the percentage of Tunel^+^ cells in all groups.
C: Representative Hsp47 (green) and Vimentin (red) immunofluorescence micrographs of on the kidney sections of uIRI mouse model in all groups. Nuclei were counterstained with DAPI (blue). Scale bar = 50 μm.
D: The bar charts showing the percent of Hsp47^+^ cells and Vimentin^+^ of uIRI mouse model in all groups, respectively.
*p < 0.05; **p < 0.01; ***p < 0.001.
**Figure S12:** Silencing of Areg Blocks BMDCs‐Driven Renal Fibrosis in a Mouse Model of IRI.
A: Experimental scheme. CD11c‐DTR mice received diphtheria toxin (DT) on days −3 and −1 to deplete endogenous CD11c⁺ DCs, followed by adoptive transfer of BMDCs transfected with control siRNA (siNC) or Areg‐specific siRNA (siAreg) on day 0 and then subjected to unilateral IRI. Kidneys were harvested on day 14.
B: AREG knockdown efficiency in BMDCs verified by Western blot.
C: Representative images of Masson trichrome, Picrosirius Red, α‐SMA immunohistochemistry and collagen I immunohistochemistry staining. Scale bars, 50 μm.
D: Quantification of fibrotic area by Masson trichrome staining.
E: Quantification of Sirius Red‐positive area. (F) Quantification of α‐SMA‐positive area and collagen I‐positive area.
*n* = 5 mice per group. Data are mean ± SEM. **p < 0.01; ***p < 0.001 by one‐way ANOVA with Tukey's post hoc test.

**Table S1:** Patients Information.

**Table S2:** The surgery time and blood loss between patients with IRI modelling and regular RN patients.

**Table S3:** Evidence on the Impact of Ischemia Duration on Renal Function after Partial Nephrectomy.

**Table S4:** Marker genes of 25 cell clusters.

**Table S5:** GSEA Result of epithelial cells.

**Table S6:** Bulk RNA sequence GSEA rasults of epithelial cells.

**Table S7:** GSEA Result of HK2 cells.

**Table S8:** GSVA Results of HK2 cells.

## Data Availability

The data that support the findings of this study are available on request from the corresponding author. The data are not publicly available due to privacy or ethical restrictions.
