# Supplementary material for: Targeting a Novel AREG + Dendritic Cell Alleviates Renal Fibrosis Induced by Ischemia–Reperfusion Injury
Source: Cell Prolif. 2026 Oct 5:e70287. Online ahead of print. doi: 10.1111/cpr.70287 (PMC13638235; doi:10.1111/cpr.70287)
Supplement: Supplementary file 1 [file CPR-9999-e70287-s003.docx]

**Table S3. Evidence on the Impact of Ischemia Duration on Renal Function after Partial Nephrectomy.**

| **First Author (Year)** | **PMID** | **Study Design** | **Sample Size** | **Ischemia Type & Duration** | **Follow-up Duration** | **Primary Outcome(s)** | ***Association of Ischemia with Renal Injury**** |
| --- | --- | --- | --- | --- | --- | --- | --- |
| Thompson  (2010) | 20825756 | Retrospective cohort | 362 | Warm ischemia, median 21 min (range 4–55); evaluated continuously and in 5-min increments | ≥30 days, long-term follow-up available | ARF, postoperative GFR <15 ml/min/1.73 m², new-onset stage IV CKD | ***Yes*** |
| Funahashi  (2014) | 25432829 | Prospective cohort | 123 | Warm ischemia: mean 24.2 min (range 8–46); Cold ischemia: mean 26.7 min (range 8–58) | 6 months | 99mTc-MAG3 renal scintigraphy: effective renal plasma flow, regional uptake | ***Yes*** |
| Volpe  (2015) | 25703575 | Systematic review | N/A (review of literature) | Warm ischemia: adverse effect when prolonged (>25 min); Cold ischemia allows longer safe ischemia | Variable (across included studies) | Postoperative renal function recovery (eGFR, parenchyma preserved) | ***Yes*** |
| Crocerossa  (2022) | 35495283 | Retrospective cohort | 457 | Warm vs. cold ischemia (technique included in model; duration not specified) | 1 year | % change in eGFR at 1 year (1YPL) | ***Yes*** |
| Lee  (2022) | 35273257 | Retrospective cohort | 104 | Cold ischemia vs. zero/selective ischemia | 12 months | % change in eGFR, parenchymal thickness and enhancement (CT) | ***Yes*** |
| Xiong  (2024) | 38000077 | Retrospective cohort | 367 | Warm ischemia (duration not specified) | Median 21.1 months (IQR 13.8–35.5) | Ipsilateral renal parenchymal volume (RPV) change; GFR fast decline (>3 ml/min/1.73 m²/year) | ***Yes*** |
| Li  (2025) | 40361128 | Meta-analysis | 4,993 | Warm ischemia; threshold harmful range 10–45 min; effect significant >25–30 min | Short-term (not specified precisely) | Postoperative eGFR, short-term renal function | ***Yes*** |
| Soranno  (2019) | 31072827 | Animal experiment | 8 mice | Warm ischemia (duration matched to human PN study; exact minutes not specified) | Short-term: 2, 6, 24 h; Long-term: 14, 28 days | Functional: GFR of injured kidney; Histology: fibrosis markers; Biomarkers in serum/urine | ***Yes*** |
| Chen  (2022) | 36465563 | Animal experiment | Not specified | Warm ischemia 0, 16, 18, 20, 22, 24, 26, 28, 30 min; contralateral nephrectomy | 24 h | Serum creatinine, histopathology (PAS), RNA-Seq molecular profiling | ***Yes*** |

***Yes = Ischemia is clearly associated with renal injury；No = Ischemia is not significantly associated with renal injury**
