# Supplementary material for: Targeting a Novel AREG + Dendritic Cell Alleviates Renal Fibrosis Induced by Ischemia–Reperfusion Injury
Source: Cell Prolif. 2026 Oct 5:e70287. Online ahead of print. doi: 10.1111/cpr.70287 (PMC13638235; doi:10.1111/cpr.70287)
Supplement: Supplementary file 2 [file CPR-9999-e70287-s005.docx]

**Table S2. The surgery time and blood loss between patients with IRI modeling and regular RN patients.**

|  | **Regular RN** | **IRI modeling** | **Significant** |
| --- | --- | --- | --- |
| Participants, n | 29 | 21 |  |
| Surgery time (min) | 149.1±47.8 | 154.9±52.9 | ns |
| Blood loss (ml) | 206.2±192.3 | 219.5±256.5 | ns |
| Electrolyte disturbance, n (%) | 0 (0%) | 0 (0%) | ns |
| Postoperative infection, n (%) | 0 (0%) | 0 (0%) | ns |
| Delayed wound healing, n (%) | 0 (0%) | 0 (0%) | ns |
| Hemorrhage or hematoma, n (%) | 0 (0%) | 0 (0%) | ns |
| Cardiovascular event, n (%) | 0 (0%) | 0 (0%) | ns |
| Reoperation, n (%) | 0 (0%) | 0 (0%) | ns |
| Mortality, n (%) | 0 (0%) | 0 (0%) | ns |

Note: All statistical results are displayed as Mean ± SD.
