# Supplementary material for: Targeting a Novel AREG + Dendritic Cell Alleviates Renal Fibrosis Induced by Ischemia–Reperfusion Injury
Source: Cell Prolif. 2026 Oct 5:e70287. Online ahead of print. doi: 10.1111/cpr.70287 (PMC13638235; doi:10.1111/cpr.70287)
Supplement: Supplementary file 3 [file CPR-9999-e70287-s007.docx]

***Fig.S1 Correlation between Ischemia Time and AIRD or Parenchymal Volume Change in Patients without Pre-existing Diseases.***

A: Ischemia time was positively correlated with the *acute ischemia-related dysfunction (AIRD) score* (R = 0.24, p = 0.001).

B: Ischemia time was also positively correlated with the percentage decrease in ipsilateral renal parenchymal volume (RPV) (R = 0.16, p = 0.007).

***Fig.S2 Additional Information on Single-cell Sequencing.***

A: The table shows three pairs of needle biopsy samples that underwent single-cell sequencing and their cell counts and survival rates.

B-E: The UMAP plots show celltypes (B), clusters origin (C), groups origin (D) and samples origin (E) by color.

***Fig.S3 Annotation and Cell States of Endothelial Subsets pre- and post-IRI.***

A-B: UMAP plot (A) of endothelial cell clusters identified based on the expression of highly variable genes and violin plots (B) showing endothelial cell marker expression in the three subclusters.

C: Dot plot shows differentially enriched pathways by GSEA in endothelial cell subsets between pre- and post-IRI.

D: PPI network for top 20 hub genes of differentially expressed genes in endothelial cells between pre- and post-IRI.

E: Violin plots showing expression of part of the hub gene in the three subclusters between pre- and post-IRI.

F: Representative ICAM1 immunofluorescence micrographs of renal endothelial cells on the kidney sections between pre- and post-IRI. Nuclei were counterstained with DAPI (blue). Scale bar=100μm.

***Fig.S4 Annotation and Cell States of LOH subsets pre- and post-IRI.***

A-B: Heatmap (A) shows the expression of marker genes in the LOH (AL) and LOH (DL). UMAP plot (B) represents the subtypes of LOH (AL) and LOH (DL) cells from the pre/post-IRI sample.

C: Feature plots showing representative gene expression for functional metabolite handling in LOH cells.

D: Violin plots showing representative gene expression for functional metabolite handling in LOH cells between pre- and post-IRI.

E: LOH (AL) pseudotime analysis.

F: Trajectory analysis shows the dynamic process of the LOH (AL) cells. RNA expression profiles change continuously along the anatomic axis in pre/post-IRI.

G-H: Two-dimensional plots (G) showing the dynamic expression of functional metabolite handling genes during the LOH (AL) cell transitions along the pseudotime in pre- (blue) and post- (red) IRI. Heatmap (H) showing the dynamic changes in gene expression along the pseudotime.

***Fig.S5 Annotation and Sell States of Collecting Duct Subsets pre- and post-IRI.***

A-B: UMAP plot (A) of collecting duct cell clusters identified based on the expression of highly variable genes and violin plots (B) showing collecting duct marker expression in the two subclusters.

C: Collecting duct cells pseudotime analysis.

D-E: Trajectory analysis shows the dynamic process of the collecting duct cells. RNA expression profiles change continuously along the anatomic axis in pre/post-IRI (D) and collecting duct subclusters (E).

F: Trajectory analysis shows RNA expression change of LCN2 along the anatomic axis.

G-H: Two-dimensional plots (G) showing the dynamic expression of inflammatory and injury genes during the collecting duct cell transitions along the pseudotime in pre- (blue) and post- (red) IRI. Heatmap (H) showing the dynamic changes in gene expression along the pseudotime.

***Fig.S6 Analyses of Stromal Cells.***

A-B: UMAP plot (A) of stromal cell clusters identified based on the expression of highly variable genes and violin plots (B) showing stromal cell marker expression in the three subclusters.

C: Collecting duct cells pseudotime analysis.

D-E: Trajectory analysis shows the dynamic process of the fibroblast and myofibroblast. RNA expression profiles change continuously along the anatomic axis in pre/post-IRI (D) and the two subclusters (E).

F-G: Two-dimensional plots(F) showing the dynamic expression of inflammatory and hypoxia genes during the stromal cell transitions along the pseudotime in pre- (blue) and post- (red) IRI. Heatmap (G) showing the dynamic changes in gene expression along the pseudotime.

***Fig.S7 Marker genes of renal resident dendritic cells and plasmacytoid dendritic cells***

***Fig S8. Identification and characterization of human renal CD11c⁺ dendritic cells.***

(A-B) CD11C+AREG+ DCs in Human Dignature Genes.

(C) Representative flow cytometry gating strategy for sorting CD11c⁺ DCs from human renal tissue after RIRI using conventional DC markers (CD45⁺CD11c⁺HLA-DR⁺CD14⁻).

(D) Representative multiplex immunofluorescence (mIF) images of human kidney biopsies pre- and post-IRI. Sections were stained for E-cadherin (green), Vimentin (red), CD11c (cyan), AREG (magenta), and DAPI (blue). Scale bar = 50 μm.

(E) Quantification of CD11c⁺AREG⁺ DC density (cells/mm²) in pre-IRI versus post-IRI kidneys. Data are individual values with mean ± SEM. ***p < 0.001 by paired t-test.

(F) Quantification of partial EMT area (defined as Pan-CK⁺ tubules with E-cadherin loss and Vimentin upregulation) as a percentage of total tubular area. *p < 0.05 by paired t-test.

(G) Correlation analysis between partial EMT area (%) and CD11c⁺AREG⁺ DC density (cells/mm²). Each dot represents a field of view (n=21). R = 0.44, p = 0.04.

***Fig.S9 Single-cell RNA Sequencing Analysis of Renal Dendritic Cell Subsets after uIRI.***

A–C: UMAP visualization of single-cell transcriptomes showing cluster origins (A), group distribution (B, sham vs. uIRI), and annotated cell types (C).

D: Dot plot depicting canonical marker gene expression across major renal cell populations.

E: Dot plot showing expression levels of Itgax and Areg among different cell types, with Areg highly expressed in dendritic cells (DCs).

F: Violin plots showing expression of marker genes used to define dendritic cell subsets (Xcr1, Clec9a, Btla, Cd209a, Itgam, Irf4).

G: Violin plots illustrating Itgax and Areg expression within DC subsets, with Areg predominantly expressed in cDC2 cells, especially elevated after uIRI.

H–I: Validation using external datasets (GSE139107 and GSE180420) confirmed that Areg expression in dendritic cells is markedly upregulated during the early phase of ischemia–reperfusion injury, followed by a decline at later stages.

***Fig.S10 Dynamic Changes in AREG Expression in Renal CD11c⁺ Cells after uIRI.***

A: Experimental design illustrating the uIRI model and kidney tissue collection at 1 h, 3 h, 6 h, 12 h, and 24 h post-reperfusion.

B: Flow cytometry gating strategy used to identify CD45⁺CD11c⁺AREG⁺ cells from kidney single-cell suspensions.

C–D: In CD45⁺CD11c⁺ cells, AREG expression was significantly increased as early as 1 h after reperfusion, reaching a peak in the early phase.

E–F: In contrast, AREG upregulation in CD11c⁻ cells was detected later, becoming evident at 12 h after reperfusion, indicating a delayed expression pattern compared with CD11c⁺ cells.

**p<0.01; ***p<0.001.

***Fig.S11 Changes in renal Hsp47, Vimentin, and α-SMA expression among groups.***

A: Representative Ngal (red) and LTL (green) immunofluorescence micrographs on the kidney sections of uIRI mouse model in all groups. Nuclei were counterstained with DAPI (blue). Scale bar=100μm. The bar charts showing the percentage of Ngal+ cells in all groups.

B: representative images of TUNEL assay of on the kidney sections of uIRI mouse model in all groups. Nuclei were counterstained with DAPI (blue). Scale bar=50μm. The bar charts showing the percentage of Tunel^+^ cells in all groups.

C: Representative Hsp47 (green) and Vimentin (red) immunofluorescence micrographs of on the kidney sections of uIRI mouse model in all groups. Nuclei were counterstained with DAPI (blue). Scale bar = 50μm.

D: The bar charts showing the percent of Hsp47^+^ cells and Vimentin^+^ of uIRI mouse model in all groups, respectively.

*p<0.05; **p<0.01; ***p<0.001.

***Figure S12.*** ***Silencing of Areg Blocks BMDCs-Driven Renal Fibrosis in a Mouse Model of IRI.***

A: Experimental scheme. CD11c-DTR mice received diphtheria toxin (DT) on days −3 and −1 to deplete endogenous CD11c⁺ DCs, followed by adoptive transfer of BMDCs transfected with control siRNA (siNC) or Areg-specific siRNA (siAreg) on day 0, and then subjected to unilateral IRI. Kidneys were harvested on day 14.

B: AREG knockdown efficiency in BMDCs verified by Western blot.

C: Representative images of Masson trichrome, Picrosirius Red, α-SMA immunohistochemistry, and collagen I immunohistochemistry staining. Scale bars, 50 μm.

D: Quantification of fibrotic area by Masson trichrome staining.

E: Quantification of Sirius Red-positive area. (F) Quantification of α-SMA-positive area and collagen I-positive area.

n = 5 mice per group. Data are mean ± SEM. **p<0.01; ***p<0.001 by one-way ANOVA with Tukey’s post hoc test.
