# Supplementary material for: Targeting a Novel AREG + Dendritic Cell Alleviates Renal Fibrosis Induced by Ischemia–Reperfusion Injury
Source: Cell Prolif. 2026 Oct 5:e70287. Online ahead of print. doi: 10.1111/cpr.70287 (PMC13638235; doi:10.1111/cpr.70287)
Supplement: Supplementary file 4 [file CPR-9999-e70287-s009.docx]

***Supplemental Methods***

***Ethics & Surgical Procedure***

Risk–benefit evaluation. This study was reviewed and approved by the Institutional Review Board of Sun Yat-sen University Cancer Center (approval No.: B2023-162-01). The ischemia/reperfusion procedure was performed only on kidneys already scheduled for radical nephrectomy. Because the kidneys were to be removed for oncological reasons, the temporary vascular clamping and biopsy collection did not pose additional risks to patient outcomes. The potential scientific benefit of elucidating the mechanisms of ischemia-reperfusion injury was considered to outweigh the minimal procedural risk. All patients provided written informed consent after detailed discussion of the study purpose, procedures, and potential risks.

Intraoperative safety measures. During the ischemia/reperfusion modeling, the renal artery was clamped under direct vision for a strictly limited duration (median 46 minutes; IQR 32-55), followed by reperfusion range from 19 to 33 minutes. Vital signs, urine output, and hemodynamic parameters were continuously monitored by the anesthesiologist. The procedure was performed by an experienced surgical team under standard sterile and anesthetic conditions. No deviation from the planned surgical protocol occurred.

Postoperative adverse-event monitoring. Postoperative monitoring included standard assessments of renal function, urine output, blood pressure, and serum electrolytes, as well as routine imaging and laboratory tests. No ischemia-related complications or unexpected adverse events occurred in any patient during hospitalization or follow-up (**Table S2**).

***Initial sample processing for scRNA-seq***

We strictly followed the methods described by Young et al.[1] in preparing our own single cell suspension. Briefly, tissues were rinsed in HBSS (Gibco) until there was no visible blood, then cut into approximately 1 mm^3^ tissue pieces and digested for 30 min at 37°C with agitation in a digestion solution containing 25µg/ml Liberase TM (Roche) and 50µg/ml DNase (Sigma) in RPMI (Gibco). Sample dissociation solutions were filtered through a 70-μm cell strainer, washed with PBS and live cells were enriched using a Dead Cell Removal Kit (Miltenyi Biotec) according to the manufacturer's instructions. The single cell suspension was stained with 0.4% trypan blue (Thermo Fisher Scientific) to examine the concentration of live cells. The cell suspensions were immediately used for single cell library preparation.

***Library preparation and sequencing.***

Cellular suspensions were loaded on a 10X Genomics GemCode Single-cell instrument that generates single-cell Gel Bead-In-EMlusion (GEMs). Libraries were generated from the cDNAs with 10X Genomics Chromium Single-Cell 3′ kit (V3). The libraries were then pooled and sequenced on an Illumina NovaSeq 6000 system by LC-Bio Technology Co., Ltd, (Hangzhou, China).

***scRNA-seq data processing***

Raw sequencing data were analyzed following the standard Chromium Cell Ranger pipeline (version 7.1.0) to align raw sequence reads to the reference genome (Ensembl，v105) using STAR. Count matrices from different batches were merged, integrated, and processed using the Seurat R package (version 4.2.0)[2]. For quality control, cells with mitochondrial gene percentages less than 30% and detected genes between 200 and 7000 were retained. During normalization (scale. factor =10,000) we also removed low-quality cells by setting vars.to.regress = c("percent.mt", "nFeature_RNA") in this step. Highly variable genes (top 2000) were extracted using the Seurat function FindVariableGenes. Principal component analysis (PCA) was then performed on the highly variable genes, and significant PCs (top 20) were selected for dimension reduction. Clusters were found using the FindClusters function (dims. use = 1:20, resolution = 0.8). UMAP analysis was used for dimension reduction and visualization of gene expression. FindAllMarkers() and FindMarkers() functions with default parameters were used on the normalized SCT data to identify DEGs. The visualization of gene expression by scatter plots, dot plots, violin plots, and volcano plots was implemented by the functions "FeaturePlot()", "DotPlot()", "VlnPlot()" in the Seurat package, "EnhancedVolcano()" in the EnhancedVolcano package and "ggplot()" in the ggplot2 package. The UMAP plots were plotted using the DimPlot() function.

***Pseudotime trajectory analysis***

For trajectory analysis, we used the R package Monocle3 (version 1.3.1).[3] The metadata from the integrated Seurat object and the top 2000 variable genes from the integrated assay were loaded into Monocle. The epithelial cells were reclustered by monocle and then trajectory analysis was performed using the learn_graph function. We used the graph_test function to derive DEGs from each cluster and genes with q-value < 0.01 and morans_I > 0.2 were used to order the cells in the pseudotime analysis. Once the cell trajectories were constructed, the genes that varied according to pseudotime were identified using the 'graph_test' function in Monocle3 and used to perform GO enrichment analysis.

***Pathway analysis***

We performed gene set enrichment analysis (GSEA) to understand the pathways of the epithelial cell using an R package, clusterProfiler R package (version 4.6.2).[4] We evaluated pathway enrichment in the major functional databases: Hallmark Gene Sets. Specifically, for the GSEA analysis, the p-value cutoff was set to 0.05 and the minGSSize was set to 5 in the 'GSEA()' function to ensure the statistical significance of each enrichment item.

***Cell-cell interaction analysis***

To investigate potential interactions between different cell types, we performed cell-cell interaction analysis using the CellChat R package (version 1.6.1), which integrates a curated repository of ligand-receptor (L-R) pairs and employs a statistical framework.[5] Networks of interactions between cell clusters were examined.

***Histology***

The kidney biopsies were fixed in 4% paraformaldehyde at 4°C for 24 hours. The specimens were then dehydrated, cleared in xylene, and embedded in paraffin wax. The embedded specimens were serially sectioned at 5 μm using a rotary microtome (Leica, Solms, Germany). The kidney biopsy slices were stained with hematoxylin and eosin (HE), periodic acid Schiff (PAS), or Masson's trichrome (Masson) to reveal the presence of fibrosis. Acute tubular injury was scored on a 0–5 scale according to Parekh et al.[6]: 0, normal; 1, mild focal swelling/blebs; 2, moderate–severe swelling in minority; 3, severe swelling in majority; 4, necrosis in minority; 5, necrosis in majority.

***Immunohistochemistry (IHC) and multiplex immunofluorescence (mIF) staining***

For IHC, after deparaffinization, slides were hydrated in alcohol, and endogenous peroxidase activity was quenched in 10% hydrogen peroxide for 30 min. Antigen epitope retrieval was induced by microwave heating. Kidney sections (4 µm) were blocked with 10% goat serum (Boster, China, Cat#AR0009) for 60 min and incubated with primary antibodies overnight at 4°C. After three 5-minute washes in phosphate-buffered saline (PBS), the sections were further incubated with goat anti-mouse / rabbit IgG polymer (Cat#PV-6000, ZSGB-BIO, Beijing) and incubated at room temperature for 1 hour. The signal was detected using DAB (ZSGB-BIO, Beijing) according to the manufacturer's protocol.

For mIF, primary antibodies were used to perform multiplex IF staining with the multiplex immunohistochemistry/immunofluorescence staining kits (absin, Shanghai, China, Cat# abs50014) according to the manufacturer's instructions. The paraffin-embedded sections (4 µm) were blocked with 10% goat serum (Boster, China, Cat#AR0009) for 60 min and incubated sequentially with the primary antibodies for 60 min at room temperature (RT). After washing, the sections were probed with HRP-conjugated anti-rabbit or anti-mouse IgG for 10 min at RT and reacted with fluorophore-conjugated tyramine molecules (PPD 520, PPD 570, PPD 620 or PPD700) for 10 min. At the end of each staining cycle, the primary and secondary antibodies were washed with PBS. All sections were then counterstained with DAPI for 5 minutes. The number of positive cells and the IHC score were calculated using HALO software (Indicalab, New Mexico, USA).

***Western Blotting***

Cells were lysed in RIPA buffer (Beyotime, Cat#P0013B) and protease cocktail inhibitor Ι (Calbiochem, San Diego, CA). Cell lysates were separated by SDS-PAGE (10% or 12.5% polyacrylamide, EpiZyme, Shanghai) and transferred to polyvinylidene difluoride membranes (PVDF, Roche). Blots were blocked in TBST containing 5% skimmed milk and incubated with primary antibodies overnight at 4˚C. The membranes were washed with TBST 4×10 min and then incubated with secondary antibodies (Beyotime, Shanghai, China) for 1 h at room temperature. After three further washes with TBST, immunoreactive bands were detected using enhanced chemiluminescence (Tanon, Shanghai, China), followed by exposure to the ChemiDoc Touch Imaging System (BIO-RAD, Hercules, CA).

***Cell culture***

HK-2 cells were cultured in DMEM/F-12 supplemented with 10% fetal bovine serum (FBS) and 1% penicillin-streptomycin. Cells were maintained at 37°C in a humidified incubator with 5% CO₂. The culture medium was replaced every 2 days, and cells were passaged at 80–90% confluence using trypsin-EDTA.

***Pharmacological inhibitor treatment***

To investigate the involvement of the AREG–EGFR–ERK signaling axis in DC-induced EMT, HK-2 cells were treated with the selective EGFR inhibitor PD153035 (5 μM) or the selective ERK1/2 inhibitor ASN007 (1 μM) for 48 h. For co-culture experiments, inhibitors were added to the lower chamber concurrently with the seeding of CD11c⁺ DCs into the upper chamber and maintained throughout the 24 h co-culture period. For recombinant AREG stimulation assays, HK-2 cells were pre-treated with PD153035 (5 μM) or ASN007 (1 μM) for 1 h prior to the addition of recombinant human AREG protein, followed by a further 48 h incubation. DMSO served as vehicle control at an equivalent final concentration (<0.1%). The concentrations and exposure durations were selected based on established in vitro protocols for these tool compounds[7, 8].

***Co-culture experiments***

Co-culture experiments were performed by seeding HK-2 cells (5 x 104) in the lower chamber and DCs (1.5 x 105) in the upper chamber of a 6-well trans-well apparatus with 0.4 μm pore size (Corning Incorporated). The co-cultured cells were subjected to further analysis after 24h of co-culture.

***Differentiation of THP-1 Cells into mDCs***

For differentiation of THP-1 cells into mDCs 2 × 10^6^ cells/mL in 20 mL serum-free RPMI supplemented with 1% PenStrep and 0.05 mM 2-mercaptoethanol was placed into a T75 flask. For differentiation according to ng/mL cytokine concentrations, the following concentrations were added: 100 ng/mL rhGM-CSF, 200 ng/mL rhIL-4, 20 ng/mL TNF-α, and 200 ng/mL ionomycin[9]. The cells were cultivated for 72 h at 37 °C, 5% CO_2_.

***Mouse***

The animal experiment was approved by the Animal Research Committee of Sun Yat-sen University Cancer Center (Approval No.: L025504202404010). Male C57BL/6 mice (4 weeks old) were obtained from the Guangdong Medical Laboratory Animal Center (Foshan, China). All mice were housed under a 12:12-hour light/dark cycle, with a constant temperature of approximately 25°C and a humidity range of 35% to 75%. Mice were euthanized by cervical dislocation. All surgical procedures were performed under isoflurane anesthesia, with measures taken to minimize animal suffering.

***IRI mouse models and in vivo gene transfer***

IRI surgery was performed as previously described.[10] Briefly, mice were anesthetized by inhalation of 3% isoflurane (oxygenated). The dorsal skin was incised, and for unilateral IRI (uIRI), the left kidney was exposed and the renal pedicle was clamped with a vascular clip for 30 minutes. Mice were maintained at a constant body temperature of 37°C. The sham group only underwent anesthesia and muscle incision without renal pedicle clamping.

The empty expression vector and plasmid vector containing mouse PLKO.1-shAreg were purchased from Ruibiotech Company (Guangzhou, China). Plasmid DNA was administered into mice using a hydrodynamic-based gene transfer technique, which involves the rapid injection of a large volume of DNA solution through the tail vein. Specifically, 20 µg of plasmid DNA was diluted in 1.8 ml saline and injected into the mouse circulation via the tail vein within 5–10 seconds.[11] Control group mice received 20 µg of the empty vector in the same manner. The plasmid was injected two days before ischemia-reperfusion, and mice were euthanized at 2 hours, 1 day, and 14 days post-ischemia-reperfusion. Kidney tissue samples were collected for analysis (Fig 6A). The target sequences of sh-Areg are as follows: GGTCTTAGGCTCAGGCCATTA.

***DTR-CD11c transgenic mice and dendritic cell depletion***

To selectively deplete CD11c⁺ dendritic cells (DCs) in vivo, CD11c-DTR (diphtheria toxin receptor) transgenic mice were used. Mice received intraperitoneal injections of diphtheria toxin (DTX; Aladdin, #D381867, China) at a dose of 0.1 μg per mouse, administered twice within three days prior to IRI surgery[12]. This treatment induces transient ablation of CD11c⁺ DCs. Control littermates (DTR-negative) received an equal volume of sterile PBS. After DTX administration, efficient depletion of renal CD11c⁺ DCs was verified by flow cytometry and immunofluorescence staining. IRI was then performed as described above, and kidney samples were collected at 2 hours, 1 day, and 14 days post-reperfusion for subsequent analysis.

***Adoptive transfer experiment***

Bone marrow was isolated from wild-type C57BL/6 mice and differentiated into bone marrow-derived dendritic cells (BMDCs) using GM-CSF (20 ng/mL) for 7 days. On day 6, BMDCs were transfected with 50 nM Areg-specific siRNA (GGTCTTAGGCTCAGGCCATTA; GCAGTAGTAGCTGTCACTATC; GAAACGATACTTCAGGGAATA) using Lipofectamine RNAiMAX (Thermo Fisher) for 24 h. Knockdown efficiency was confirmed by qPCR and ELISA (>70% reduction, Figure SX).

CD11c-DTR mice received intraperitoneal injections of diphtheria toxin (DTX; Aladdin, #D381867, China) at a dose of 0.1 μg per mouse on days −3 and −1 to deplete endogenous CD11c⁺ cells[8, 12]. DTR-negative littermates received an equal volume of sterile PBS as non-depleted controls. Efficient depletion of renal CD11c⁺ DCs was verified by flow cytometry and immunofluorescence staining prior to adoptive transfer.

On day 0, 1 × 10⁶ transfected BMDCs were injected via tail vein, followed by unilateral ischemia-reperfusion injury (uIRI) surgery 2 hours later. Kidneys were harvested at day 14 post-reperfusion. Renal fibrosis was assessed by Masson trichrome staining, Picrosirius Red (Sirius Red) staining, α-SMA immunohistochemistry, and collagen I immunohistochemistry. For each method, the percentage of positive area per field was quantified using ImageJ software (NIH, Bethesda, MD, USA).

***Flow Cytometry***

Single-cell suspensions of kidney were prepared as described above. For cell surface staining, single cell suspensions were incubated with antibodies for 30 min on ice. The samples were acquired on a FACS Verse instrument (BD Biosciences) and analyzed with Flow Jo version 10.8.1 (Treestar, Ashland, OR).

***Statistical analysis***

All statistical analyses were performed using GraphPad Prism 10.0 (GraphPad Software, Inc., La Jolla, California, USA). Data are represented as mean ± SEM. Statistical significance was determined using Student t-tests and one- or two-way ANOVA with Tukey post hoc test for multiple comparisons as necessary in Prism 9 (Graphpad). A P value < 0.05 was considered statistically significant.

**Reference**

1.	Young, M.D., et al., *Single-cell transcriptomes from human kidneys reveal the cellular identity of renal tumors.* Science, 2018. **361**(6402): p. 594–599.

2.	Stuart, T., et al., *Comprehensive Integration of Single-Cell Data.* Cell, 2019. **177**(7): p. 1888–1902 e21.

3.	Cao, J., et al., *The single-cell transcriptional landscape of mammalian organogenesis.* Nature, 2019. **566**(7745): p. 496–502.

4.	Wu, T., et al., *clusterProfiler 4.0: A universal enrichment tool for interpreting omics data.* Innovation (Camb), 2021. **2**(3): p. 100141.

5.	Jin, S., et al., *Inference and analysis of cell-cell communication using CellChat.* Nat Commun, 2021. **12**(1): p. 1088.

6.	Parekh, D.J., et al., *Tolerance of the human kidney to isolated controlled ischemia.* J Am Soc Nephrol, 2013. **24**(3): p. 506–17.

7.	Portelinha, A., et al., *ASN007 is a selective ERK1/2 inhibitor with preferential activity against RAS-and RAF-mutant tumors.* Cell Rep Med, 2021. **2**(7): p. 100350.

8.	Ding, L., et al., *Bone Marrow CD11c+ Cell-Derived Amphiregulin Promotes Pulmonary Fibrosis.* J Immunol, 2016. **197**(1): p. 303–12.

9.	Holken, J.M. and N. Teusch, *The Monocytic Cell Line THP-1 as a Validated and Robust Surrogate Model for Human Dendritic Cells.* Int J Mol Sci, 2023. **24**(2).

10.	Wei, Q. and Z. Dong, *Mouse model of ischemic acute kidney injury: technical notes and tricks.* Am J Physiol Renal Physiol, 2012. **303**(11): p. F1487–94.

11.	Chen, S., et al., *Tenascin-C protects against acute kidney injury by recruiting Wnt ligands.* Kidney Int, 2019. **95**(1): p. 62–74.

12.	Roquilly, A., et al., *Local Modulation of Antigen-Presenting Cell Development after Resolution of Pneumonia Induces Long-Term Susceptibility to Secondary Infections.* Immunity, 2017. **47**(1): p. 135–147 e5.
