# Supplementary material for: Targeting a Novel AREG + Dendritic Cell Alleviates Renal Fibrosis Induced by Ischemia–Reperfusion Injury
Source: Cell Prolif. 2026 Oct 5:e70287. Online ahead of print. doi: 10.1111/cpr.70287 (PMC13638235; doi:10.1111/cpr.70287)
Supplement: Supplementary file 5 [file CPR-9999-e70287-s010.docx]

**Table S1. Patients Information.**

| **No.** | **Gender** | **Age** | **BMI** | **HTN** | **DM** | **Preoperative Scr (μmol/L)** | **eGFR**  **(mL/min/1.73m^2^)** | **Ischemia time (min)** | **Reperfusion time (min)** |
| --- | --- | --- | --- | --- | --- | --- | --- | --- | --- |
| 1 | Male | 60 | 22.23 | 0 | 0 | 68.8 | 102.2 | 32 | 19 |
| 2 | Female | 63 | 24.44 | 0 | 0 | 58.3 | 98.5 | 25 | 15 |
| 3 | Male | 65 | 23.14 | 0 | 0 | 59.5 | 103.5 | 31 | 19 |
| 4 | Female | 63 | 24.44 | 0 | 0 | 58.3 | 98.5 | 47 | 28 |
| 5 | Male | 65 | 23.14 | 0 | 0 | 59.5 | 103.5 | 53 | 32 |
| 6 | Male | 65 | 18.92 | 0 | 0 | 72.5 | 97.5 | 71 | 43 |
| 7 | Female | 32 | 20.29 | 0 | 0 | 52 | 122.8 | 46 | 28 |
| 8 | Female | 56 | 28.44 | 0 | 0 | 65.4 | 94.9 | 46 | 27 |
| *9 | Female | 57 | 22.35 | 0 | 0 | 59.7 | 101.7 | 62 | 37 |
| 10 | Female | 54 | 25.85 | 0 | 0 | 68.1 | 91.6 | 48 | 29 |
| 11 | Male | 61 | 22.28 | 0 | 0 | 88.8 | 85.2 | 32 | 19 |
| *12 | Male | 59 | 21.49 | 0 | 0 | 85.6 | 90.1 | 82 | 49 |
| 13 | Male | 72 | 18.88 | 0 | 0 | 70.5 | 94.1 | 29 | 17 |
| *14 | Male | 56 | 26.75 | 0 | 0 | 82.3 | 96.2 | 57 | 34 |
| 15 | Female | 72 | 32.81 | 0 | 0 | 45.6 | 98.8 | 43 | 26 |
| 16 | Female | 70 | 21.84 | 0 | 0 | 57.5 | 94.6 | 27 | 16 |
| 17 | Female | 65 | 24.20 | 0 | 0 | 59.9 | 96.7 | 32 | 19 |
| 18 | Male | 41 | 24.62 | 0 | 0 | 77.3 | 111.0 | 50 | 30 |
| 19 | Female | 64 | 23.86 | 0 | 0 | 58.5 | 97.8 | 74 | 44 |
| 20 | Male | 72 | 21.08 | 0 | 0 | 78.4 | 91.1 | 32 | 19 |
| 21 | Female | 58 | 22.68 | 0 | 0 | 56.7 | 102.3 | 53 | 32 |

***Note:***

***** Indicates single cell and bulk RNA sequencing was performed on this patient sample.

BMI: body mass index; HTN: hypertension; DM: diabetes mellitus; Scr: serum creatinine.
